# Supplementary material for: Decoupled temperature and pressure hydrothermal synthesis of carbon sub-micron spheres from cellulose
Source: Nat Commun. 2022 Jun 24;13:3616. doi: 10.1038/s41467-022-31352-x (PMC9232491; doi:10.1038/s41467-022-31352-x)
Supplement: Supplementary file 1 — Supplementary Information [file 41467_2022_31352_MOESM1_ESM.pdf]

Supplementary Information

Decoupled temperature and pressure hydrothermal synthesis of carbon sub-micron spheres from cellulose

Shijie Yu[a,b], Xinyue Dong[c,d], Peng Zhao[a,b], Zhicheng Luo[e], Zhuohua Sun[f], Xiaoxiao Yang[a,b], Qinghai Li[a,b], Lei Wang*[c,d], Yanguo Zhang*[a,b], Hui Zhou*[a,b]

a Key Laboratory for Thermal Science and Power Engineering of Ministry of Education, Department of Energy and Power Engineering, Tsinghua University, Beijing 100084, P.R. China

b Beijing Key Laboratory of CO_2 Utilization and Reduction Technology, Department of Energy and Power Engineering, Tsinghua University, Beijing 100084, P.R. China

c Key Laboratory of Coastal Environment and Resources of Zhejiang Province (KLaCER), School of Engineering, Westlake University, Hangzhou 310024, Zhejiang

d Institute of Advanced Technology, Westlake Institute for Advanced Study, Hangzhou, Zhejiang, China

e Department of Chemical Engineering and Chemistry, Eindhoven University of Technology, Het Kranenveld 14, Helix, STW 3.48, 5612 AZ Eindhoven, The Netherlands

f Beijing Key Laboratory of Lignocellulosic Chemistry, Beijing Forestry University, No.35 Tsinghua East Road, Beijing 100083, P. R. China

**Corresponding author: wang_lei@westlake.edu.cn (LW), zhangyg@tsinghua.edu.cn (YZ), huizhou@tsinghua.edu.cn (HZ)*

This supplementary information file includes:

Supplementary Note 1. Comparison of different hydrothermal synthesis results for carbon spheres from cellulose.. 3

Supplementary Note 2. Experimental setup of the DTPH system.................................. 4

Supplementary Note 3. DTPH reaction under different pressures.................................... 6

Supplementary Note 4. Kinetics of the DTPH reaction... 15

Supplementary Note 5. DTPH reaction under different temperatures............................ 17

Supplementary Note 6. Kinetics in TGA... 29

Supplementary Note 7. ^{1}H NMR for hydrochar in H_2O and D_2O. 33

Supplementary Note 8. Methodology of sustainability assessment................................ 34

Supplementary Note 9. Results of sustainability assessment. 43

Supplementary Note 10. Comparison between DTPH carbonization and other technologies. 45

Supplementary Note 11. Effects of other parameters on the DTPH reaction................. 48

Supplementary References.. 50

Supplementary Note 1. Comparison of different hydrothermal synthesis results for carbon spheres from cellulose.

Supplementary Table 1 | Comparison between the DTPH reaction of cellulose in this study and the CTPH reactions in the literature.

	Crystallinity index (%)	Initial decomposition temperature (°C)	Initial formation temperature of carbon spheres (°C)	Complete formation temperature of carbon spheres (°C)	Pressure (MPa)	Isothermal time (h)	Yield of carbon spheres (%)	Ref.
CTPH	88[a]	210	220	230	Not given	4.0	33.5	1
CTPH	81[a]	180	200	220	Not given	24.0	~37.0	2
CTPH	Not given	200	Not given	250	2[b]	~2.2	~32.0	3
CTPH	Not given	220	Not given	Not given	Not given	0.5	-	4
CTPH	Not given	Not given	220	220	Self-generated pressure	4.0	46.1	5
CTPH	88	210	270[c]	280[c]	Self-generated pressure	0[d]	43.5	Control experiment
DTPH	88	117	150	200	20[e]	0[d]	43.5	This work

[a] Calculated by the XRD pattern in the ref.; [b] initial pressure before heating in the CTPH process; [c] obtained based on the same mass loss as the DTPH process; [d] there is no isothermal time; the total time for heating and cooling was 1.2 h; [e] constant pressure in the DTPH process.

Supplementary Note 2. Experimental setup of the DTPH system.

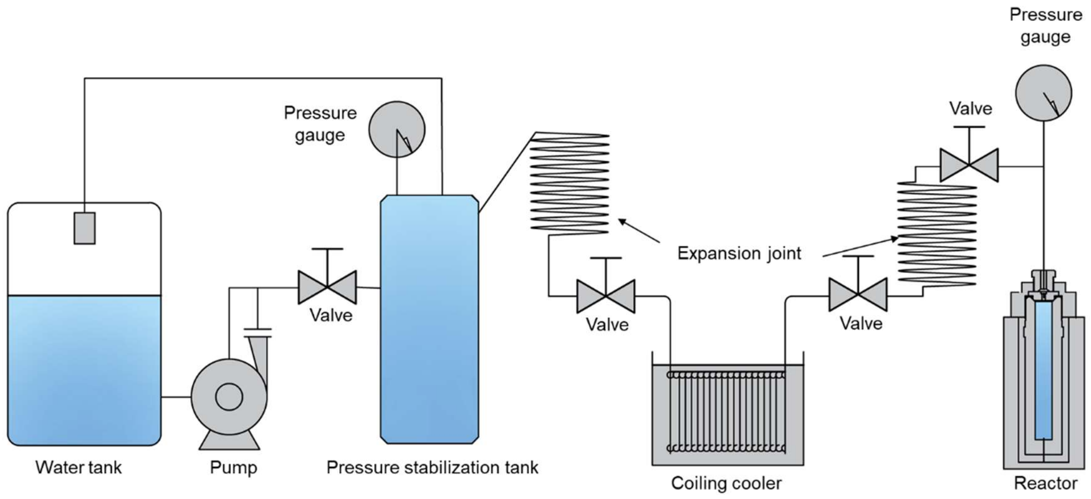

Supplementary Figure 1 | Schematic of the DTPH reaction system.

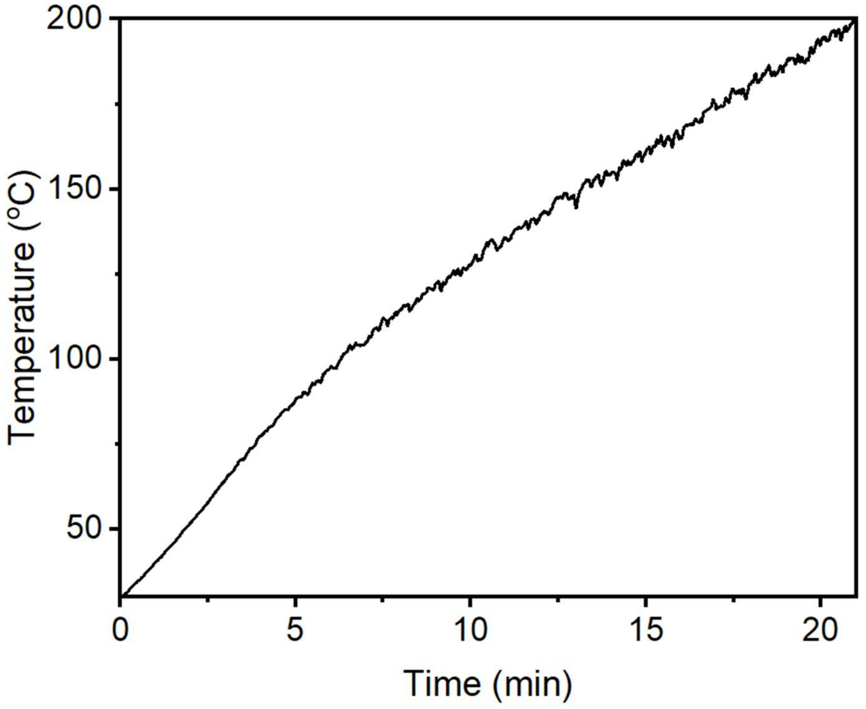

Supplementary Figure 2 | Temperature profile of the reaction.

Supplementary Note 3. DTPH reaction under different pressures.

Constant high pressure promoted the cellulose degradation from 2 to 6 MPa, and lower mass loss was achieved under higher pressures (from 6 to 20 MPa) (Supplementary Fig. 3). From 2 to 6 MPa, the main role of pressure is to break the kinetic limits and thus promote the degradation at a low temperature. Above 6 MPa, the release of small molecule products is thermodynamically inhibited, resulting in slightly higher solid yields. According to proximate analyses, elemental analyses, FTIR, and XRD, high pressures promoted the carbonization of cellulose (Supplementary Table 2 and Supplementary Figs. 4 and 5). The color of hydrothermally treated cellulose got darker with the increase of pressure (Supplementary Fig. 6), and carbon sub-micron spheres were formed (Supplementary Figs. 7–9). Moreover, the hydrothermally treated cellulose from high pressures showed much higher thermal stability during the pyrolysis and combustion in TGA (Supplementary Figs. 11 and 12).

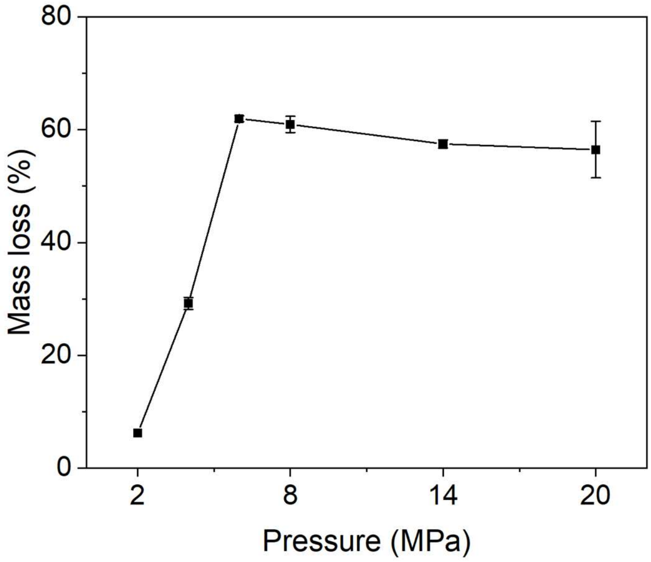

Supplementary Figure 3 | Mass loss of cellulose at 200 °C under different pressures. Error bars represent standard deviations of repeated tests.

Supplementary Table 2 | Elemental composition and proximate analysis of hydrothermally treated cellulose under different pressures at 200 °C.

Pressure (MPa)	Elemental composition (dry ash-free basis)					Proximate analysis (dry basis)		
	C (wt%)	H (wt%)	O (wt%)	O/C (atomic ratio)	H/C (atomic ratio)	Ash (wt%)	Volatile (wt%)	Fixed carbon (wt%)
Cellulose	42.5	5.6	51.9	0.92	1.58	0.0	96.3	3.7
2	43.4	6.5	50.1	0.87	1.81	0.0	96.0	4.0
8	69.1	4.6	26.3	0.29	0.80	0.0	48.5	51.5
14	70.0	4.3	25.6	0.27	0.74	0.0	50.9	49.1
20	70.7	4.5	24.8	0.26	0.77	0.0	53.8	46.2

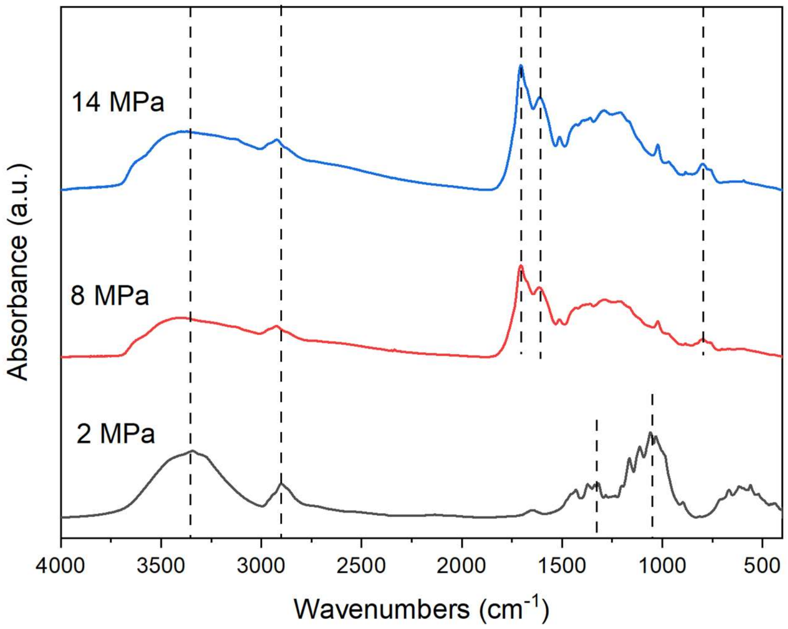

Supplementary Figure 4 | FTIR spectra of hydrothermally treated cellulose at 200 °C under different pressures.

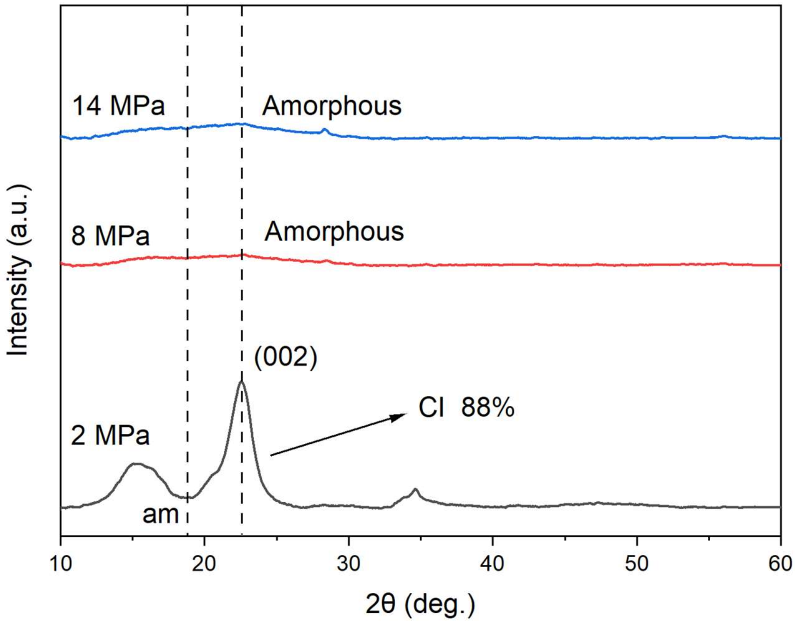

Supplementary Figure 5 | XRD patterns of hydrothermally treated cellulose at 200 °C under different pressures.

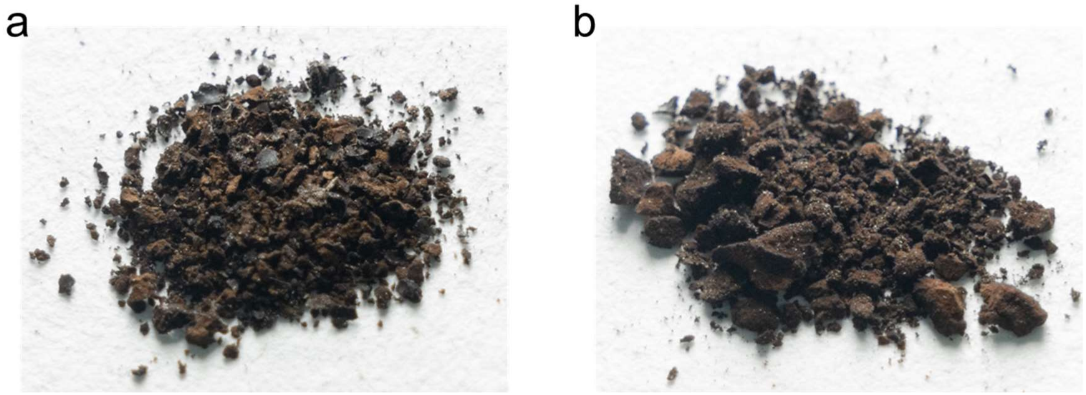

Supplementary Figure 6 | Macroscopic morphologies of hydrochar under different pressures at 200 °C. (a) 8 MPa; (b) 14 MPa.

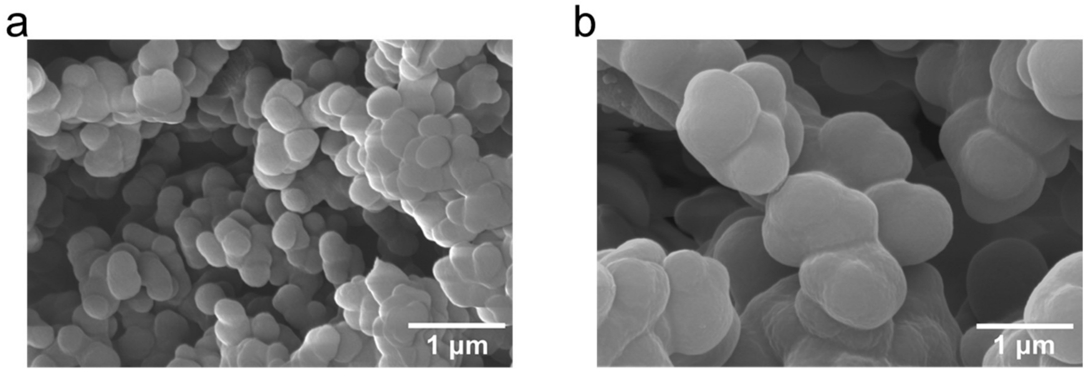

Supplementary Figure 7 | SEM of hydrochar under different pressures at 200 °C. (a) 8 MPa; (b) 14 MPa.

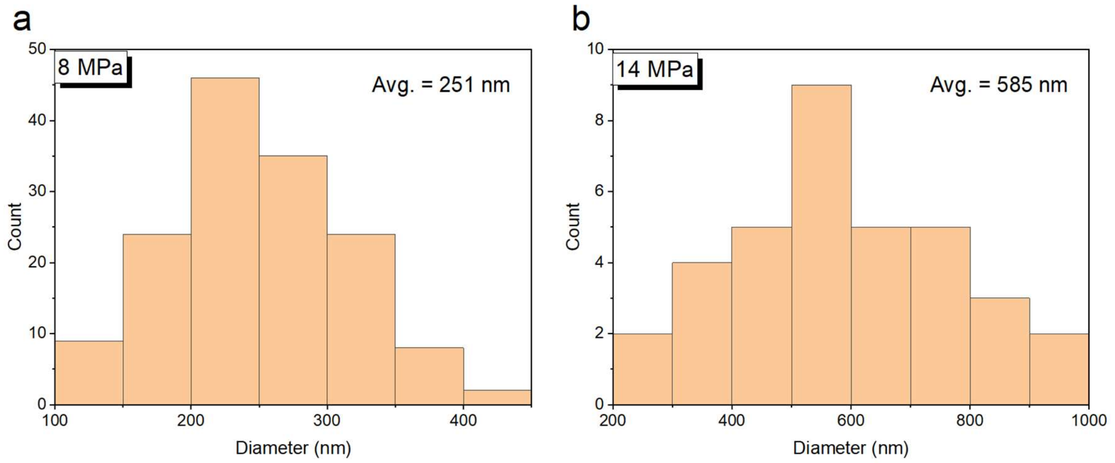

Supplementary Figure 8 | Size distribution of sub-micron spheres under different pressures at 200 °C. (a) 8 MPa; (b) 14 MPa.

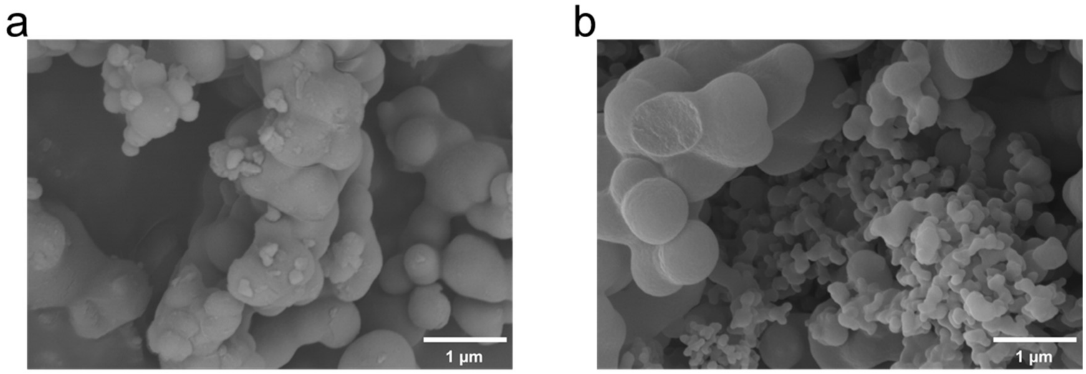

Supplementary Figure 9 | SEM of hydrochar under different pressures at 300 °C. (a) 9 MPa; (b) 20 MPa.

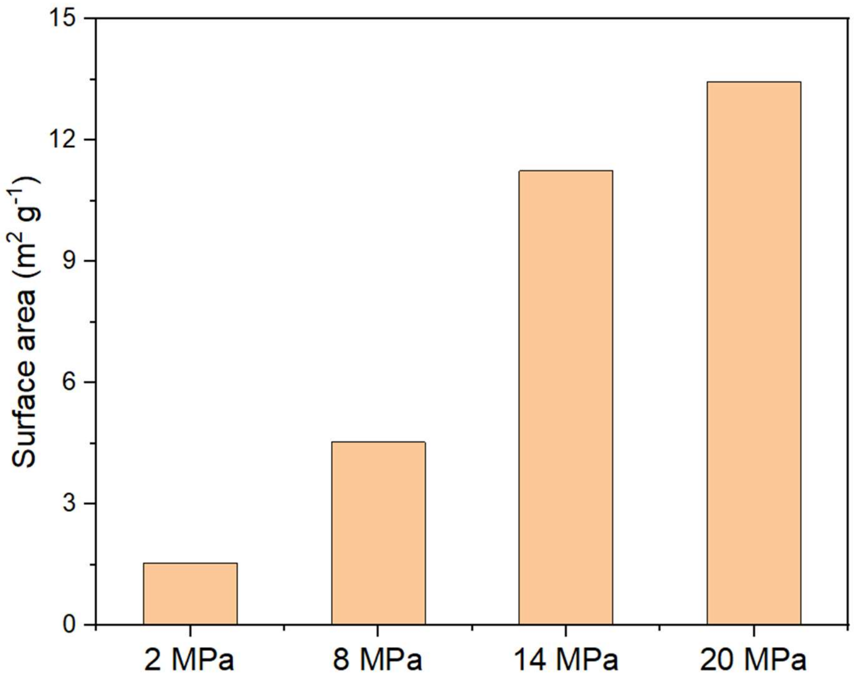

Supplementary Figure 10 | Surface area of the hydrothermally treated cellulose under different pressures at 200 °C.

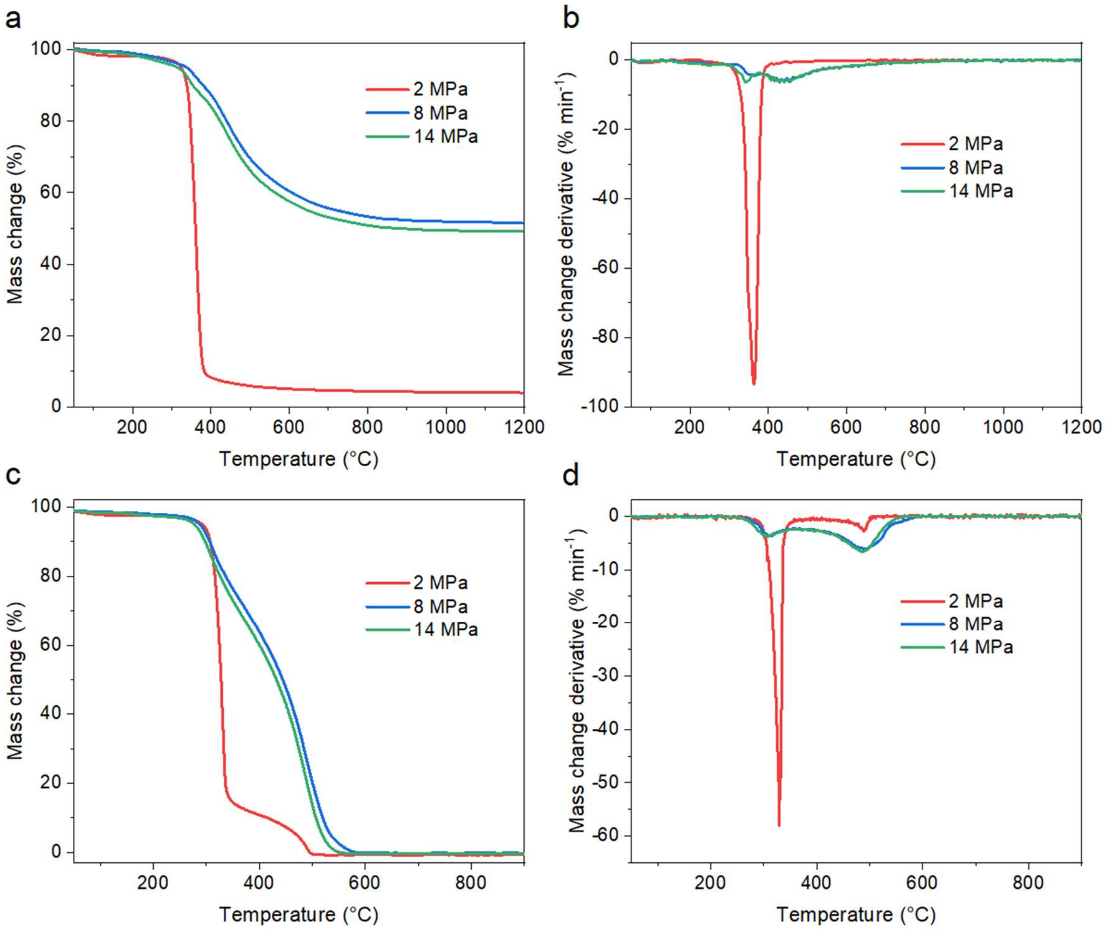

Supplementary Figure 11 | The thermogravimetric study of hydrothermally treated cellulose at 200 °C under different pressures. (a) TG curves under N_2; (b) DTG curves under N_2; (c) TG curves under air; (d) DTG curves under air.

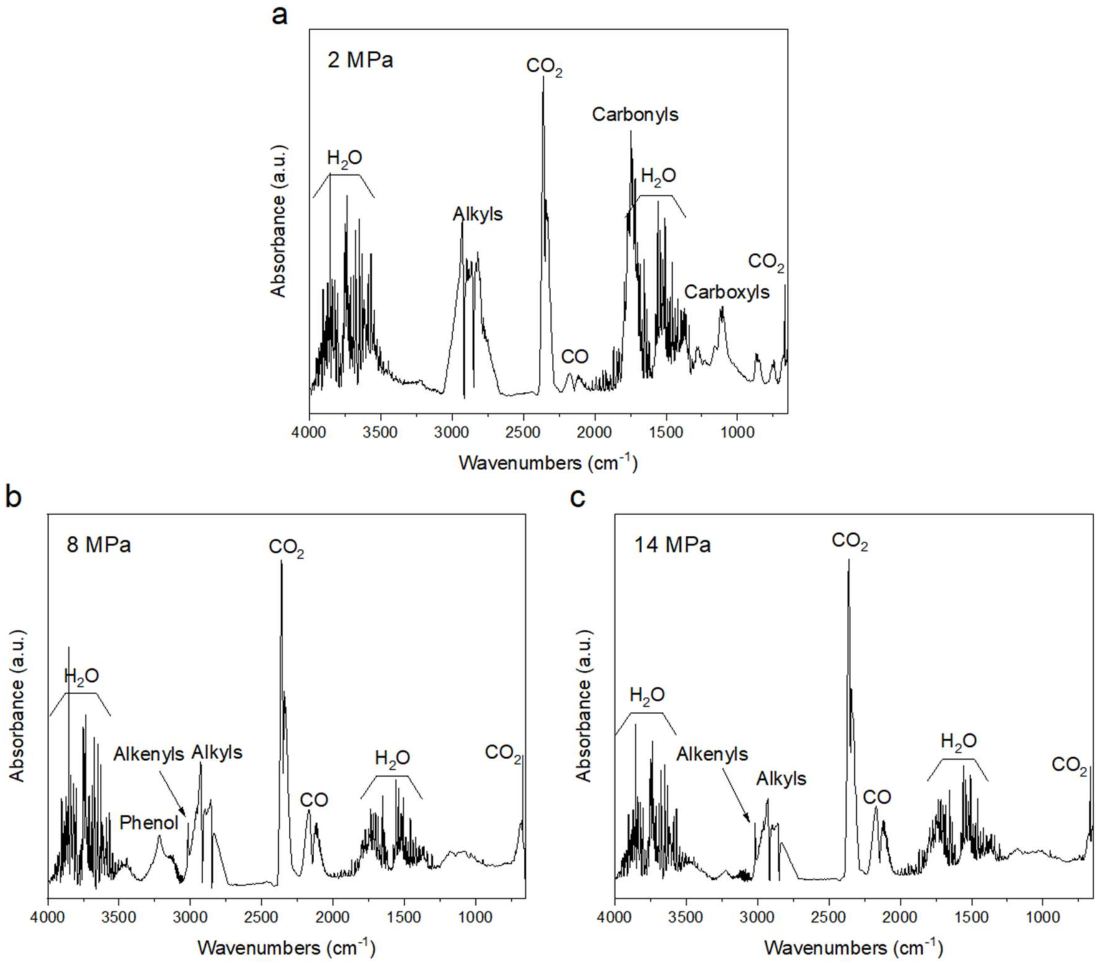

Supplementary Figure 12 | FTIR spectra of the pyrolysis products of hydrothermally treated cellulose at the maximum mass loss temperature under different pressures (200 °C). (a) 2 MPa (360 °C); (b) 8 MPa (453 °C); (c) 14 MPa (446 °C). The maximum mass loss temperatures are given in parentheses.

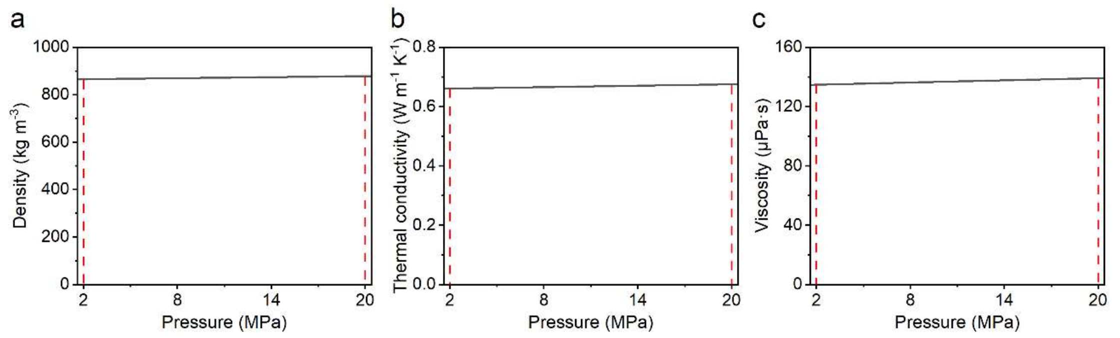

Supplementary Figure 13 | Physical properties of water under different pressures (200 °C). (a) Density; (b) thermal conductivity; (c) viscosity.

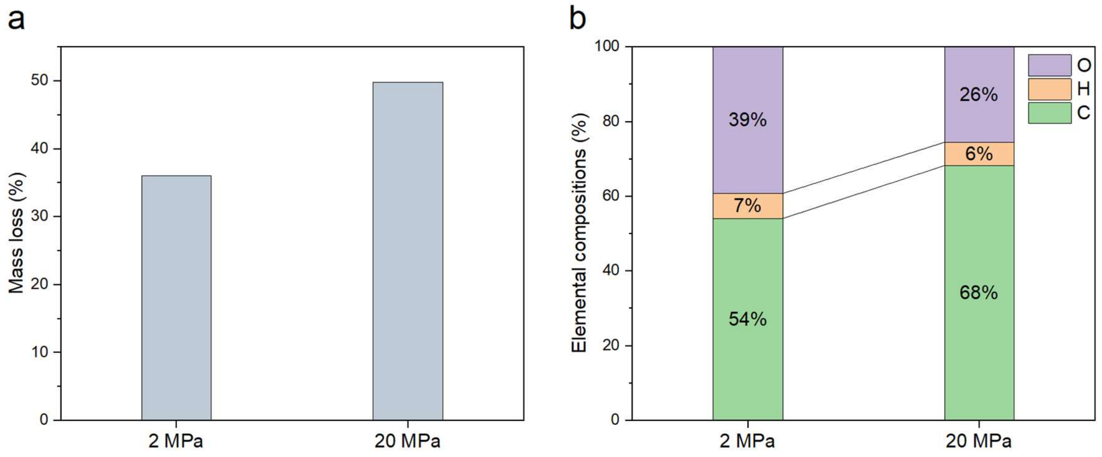

Supplementary Figure 14 | Comparison of the mass loss (a) and hydrochar elemental compositions (b) of the DTPH process of rice straw under 2 and 20 MPa (200 °C).

Supplementary Note 4. Kinetics of the DTPH reaction.

The kinetics of cellulose hydrothermal reaction were calculated with the Coats-Redfern (C-R) method[6]. In general, the hydrothermal reaction rate can be expressed using the first-order rate law[7]:

$$\frac{d\alpha}{d\tau} = k(1-\alpha)$$

where τ is the time of reaction (s); α is the conversion; k is the reaction rate constant (s^{-1}) given by:

$$k = A\exp(-\frac{E}{RT})$$

where A is the pre-exponential factor (s^{-1}); E is the apparent activation energy (kJ mol^{-1}); R is the universal gas constant (kJ mol^{-1} K^{-1}); T is the absolute temperature (K).

In hydrothermal experiments, the heating rate β is constant:

$$\beta = \frac{dT}{d\tau}$$

Combining equations above, rearranging and integrating:

$$\int_0^\alpha \frac{\mathrm{d}\alpha}{1-\alpha} = \frac{A}{\beta}\int_0^T \exp(-\frac{E}{RT})$$

Rearranging and taking logarithm:

$$\ln\left[\frac{-\ln(1-\alpha)}{T^2}\right] = \ln\left[\frac{AR}{\beta E}\left(1-\frac{2RT}{E}\right)\right] - \frac{E}{RT}$$

The kinetic parameters can be obtained by linear regression of this equation.

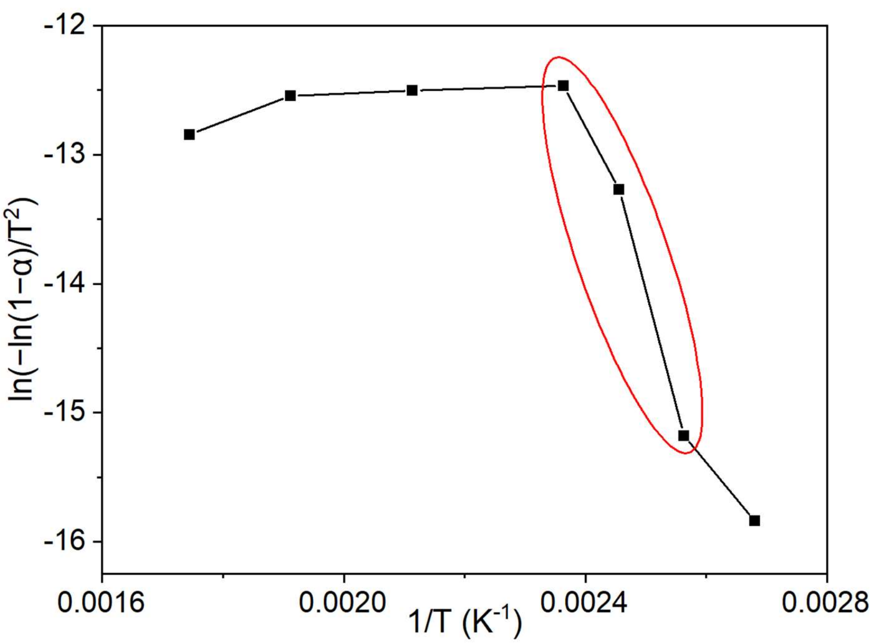

Supplementary Figure 15 | Calculation of kinetics in the DTPH process under 20 MPa.

Supplementary Note 5. DTPH reaction under different temperatures.

Proximate analysis revealed that the volatile content decreased from 96.3 wt% (100 °C) to 39.5 wt% (300 °C), and the fixed carbon increased from 3.7 wt% (100 °C) to 60.5 wt% (300 °C). The transformation from volatile-rich material into fixed carbon-rich material was consistent with the color change from white to brownish-black (Supplementary Table 3 and Supplementary Fig. 16).

Supplementary Table 3 | Elemental composition and proximate analysis of hydrothermally treated cellulose from different stages (temperatures) at 20 MPa.

Temperature (°C)	Elemental composition (dry ash-free basis)					Proximate analysis (dry basis)		
	C (wt%)	H (wt%)	O (wt%)	O/C (atomic ratio)	H/C (atomic ratio)	Ash (wt%)	Volatile (wt%)	Fixed carbon (wt%)
Cellulose	42.5	5.6	51.9	0.92	1.58	0.0	96.3	3.7
100	43.0	6.0	51.0	0.89	1.67	0.0	96.2	3.8
150	66.7	4.6	28.7	0.32	0.82	0.0	63.8	36.2
200	70.7	4.5	24.8	0.26	0.77	0.0	53.8	46.2
250	72.2	4.3	23.5	0.24	0.71	0.0	48.4	51.6
300	76.2	4.4	19.4	0.19	0.69	0.0	39.5	60.5

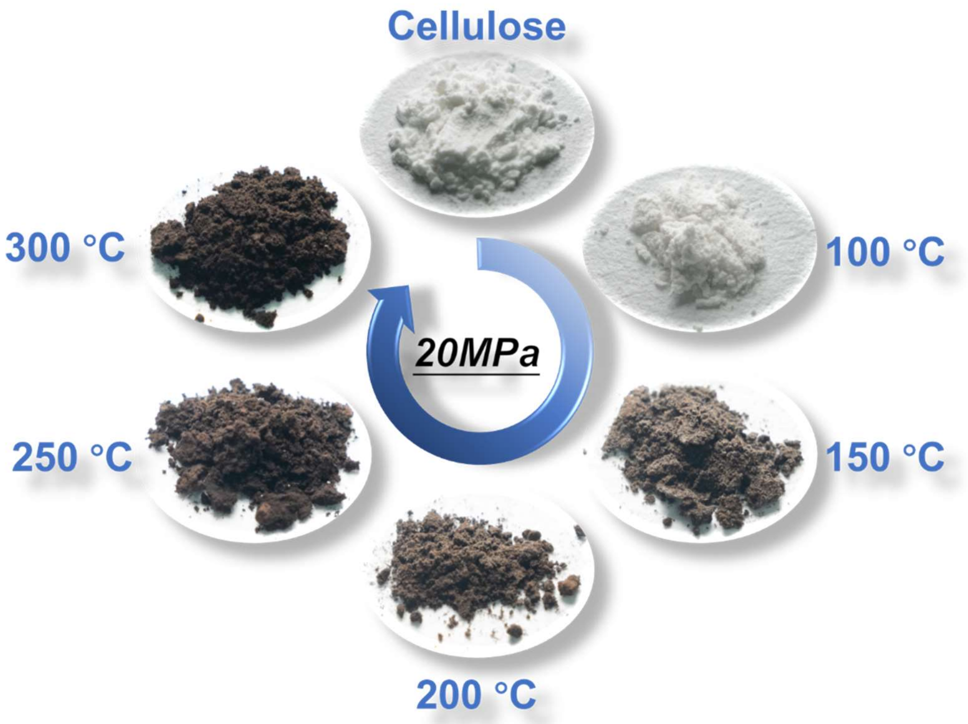

Supplementary Figure 16 | Macroscopic morphologies of hydrothermally treated cellulose from different stages (temperatures) of the hydrothermal reaction at 20 MPa.

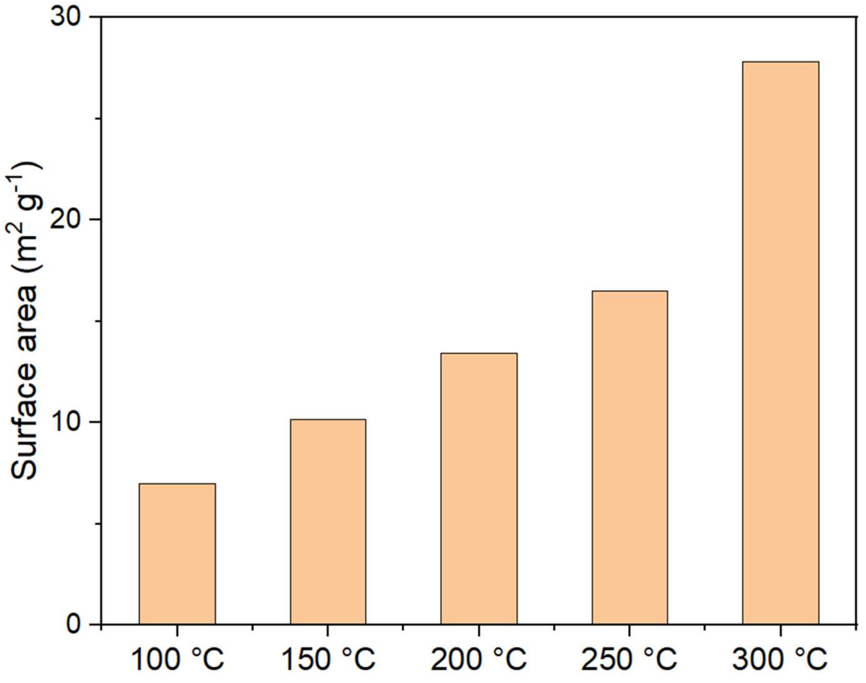

Supplementary Figure 17 | Surface area of the hydrothermally treated cellulose from different stages (temperatures) at 20 MPa.

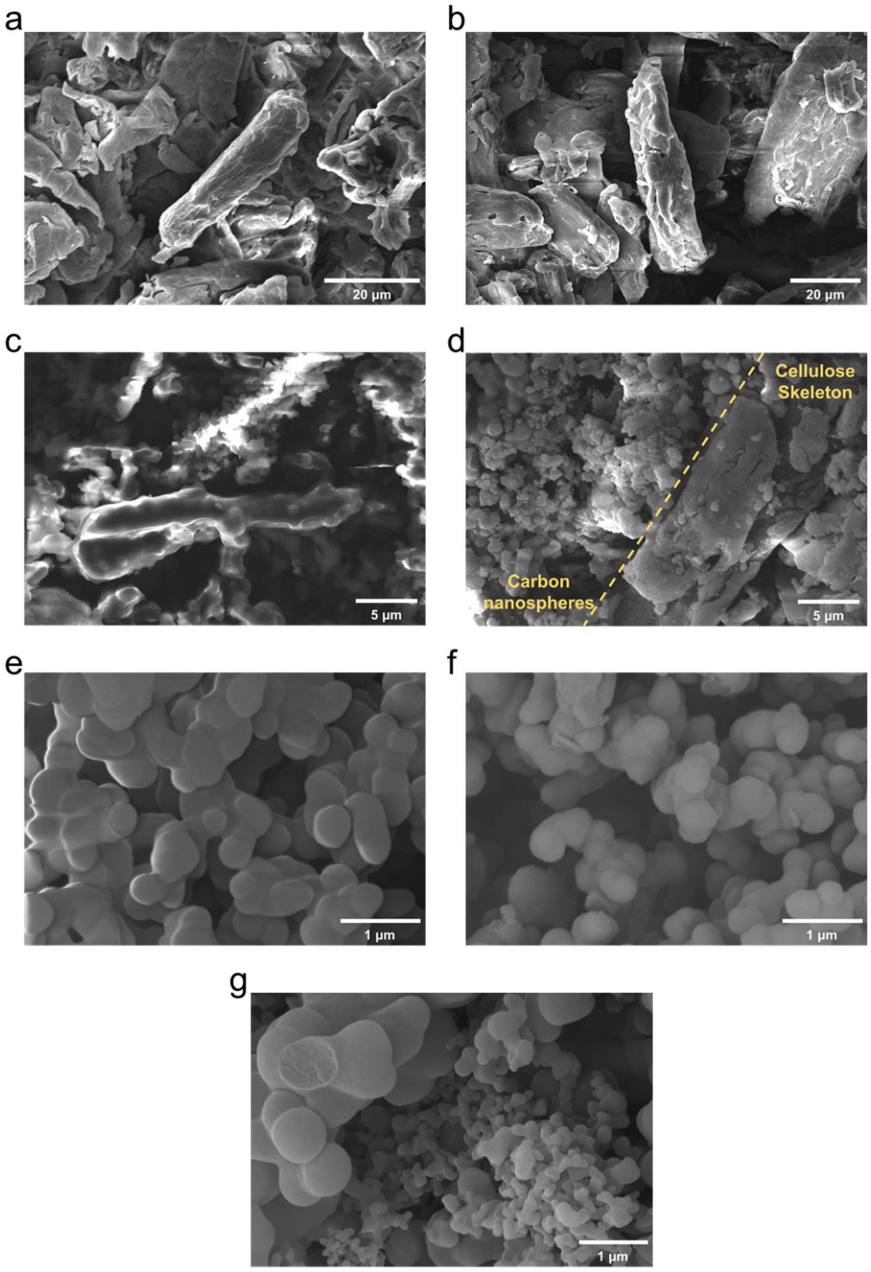

Supplementary Figure 18 | SEM of hydrothermally treated cellulose in the DTPH process (20 MPa). (a) Original cellulose; (b) hydrothermally treated cellulose from 100 °C; (c) and (d) hydrothermally treated cellulose from 150 °C; (e) hydrothermally treated cellulose from 200 °C; (f) hydrothermally treated cellulose from 250 °C; (g) hydrothermally treated cellulose from 300 °C.

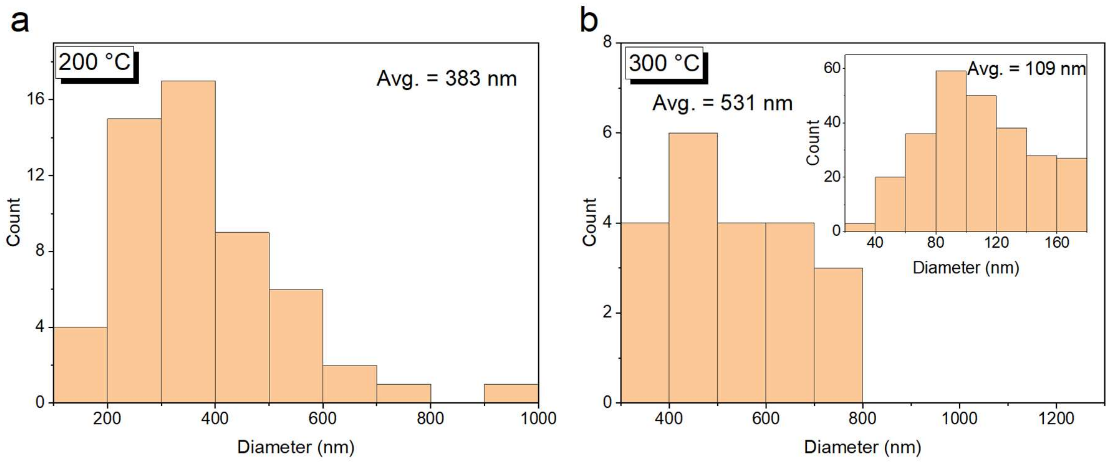

Supplementary Figure 19 | Size distribution of sub-micron spheres at different temperatures in the DTPH process under 20 MPa. (a) 200 °C; (b) 300 °C.

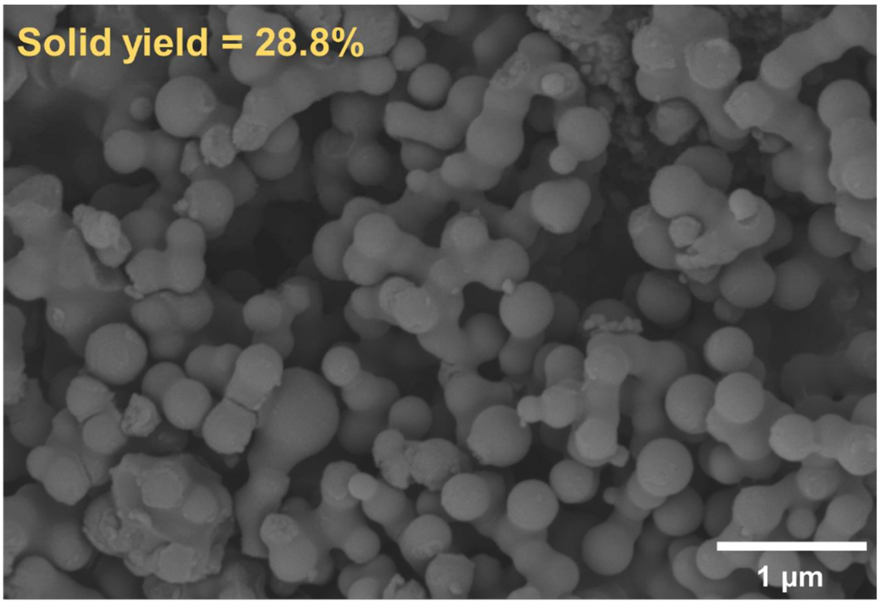

Supplementary Figure 20 | SEM of hydrochar from glucose at 300 °C and 20 MPa.

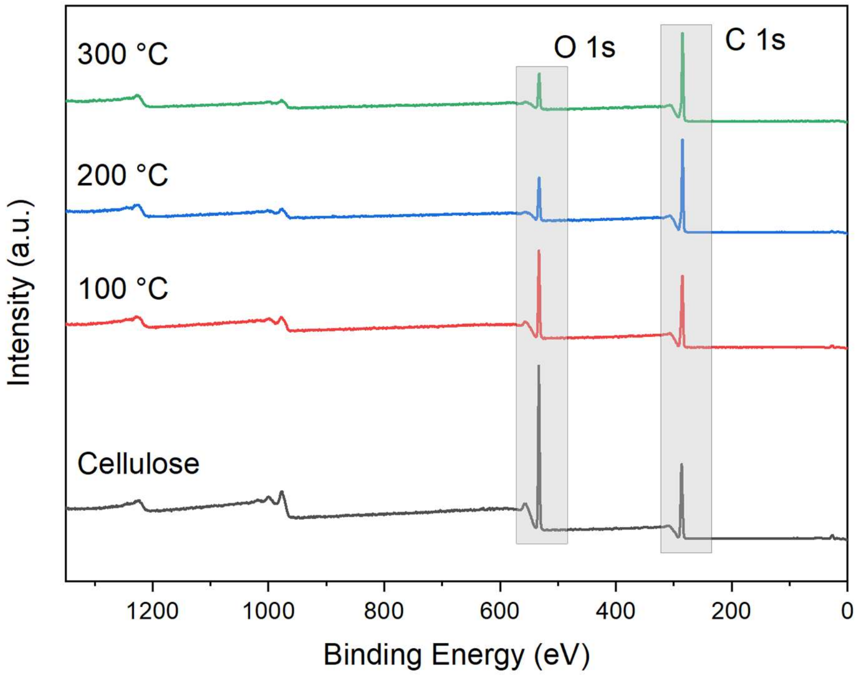

Supplementary Figure 21 | XPS survey spectra of hydrothermally treated cellulose from different stages of the hydrothermal treatment of cellulose (20 MPa).

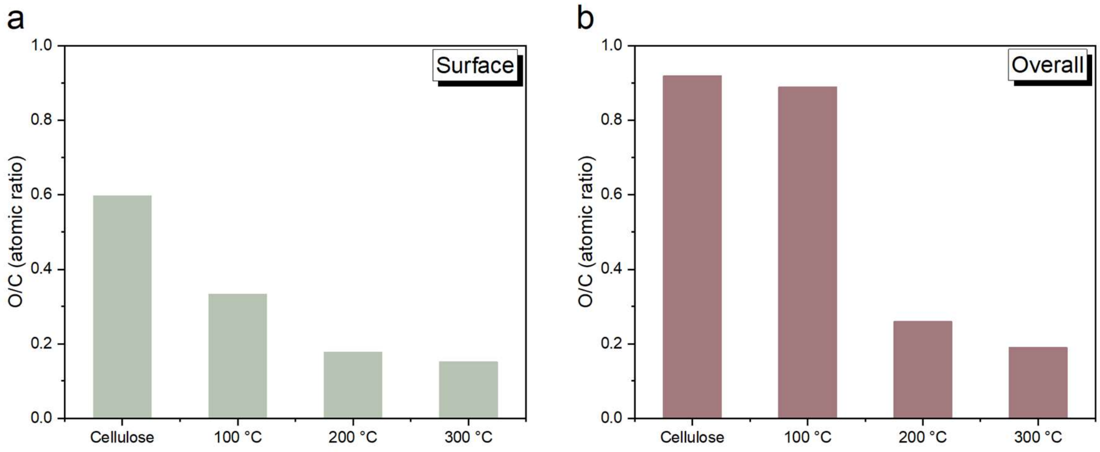

Supplementary Figure 22 **| O/C atomic ratios of hydrothermally treated cellulose from different stages of the hydrothermal treatment of cellulose. (a) Surface O/C ratio from XPS survey spectra; (b) overall O/C ratio from elemental analysis (see Supplementary Table 1 for details).**

Supplementary Table 4 | C 1*s* fitting parameters for hydrothermally treated cellulose from different stages of the hydrothermal treatment of cellulose (20 MPa).

Temperature (°C)	Species	Binding energy (eV)	FWHM (eV)	Peak shape	Amount (%)
Cellulose	C–C	285.0	1.37	GL*	21
	C–O	286.7	1.19	GL	59
	O–C–O	288.0	1.53	GL	20
100	C–C	285.0	1.25	GL	54
	C–O	286.7	1.29	GL	35
	O–C–O	288.5	1.52	GL	11
200	C–C/C=C	285.0	1.29	GL	77
	C–O	286.5	1.51	GL	15
	O–C=O	289.0	1.11	GL	8
300	C–C/C=C	285.0	1.37	GL	82
	C–O	286.6	1.49	GL	12
	O–C=O	289.0	1.10	GL	6

* GL- Gaussian/Lorentzian line shape.

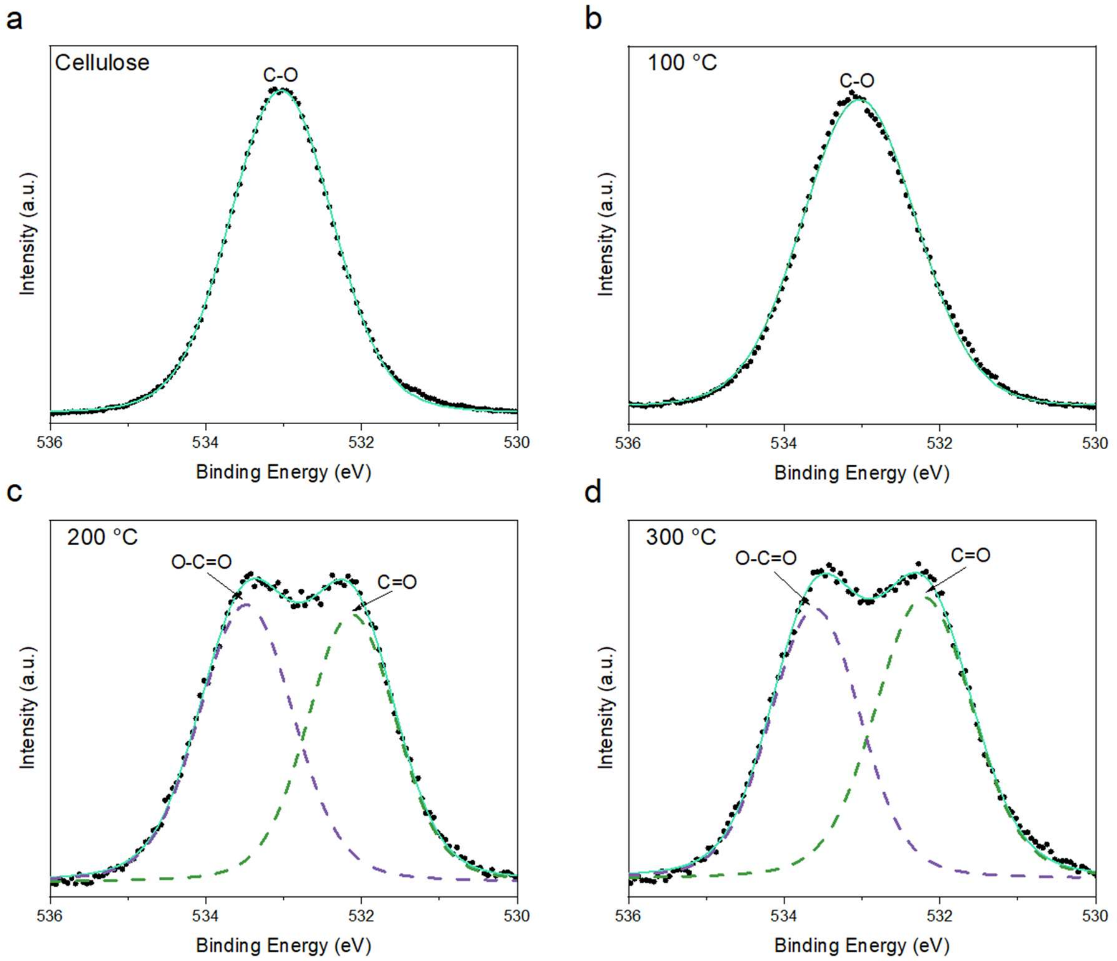

Supplementary Figure 23 | O 1*s* spectra of hydrothermally treated cellulose from different stages of the hydrothermal treatment of cellulose under 20 MPa.

Supplementary Table 5 | O 1*s* fitting parameters for hydrothermally treated cellulose from different stages of the hydrothermal treatment of cellulose (20 MPa).

Temperature (°C)	Species	Binding energy (eV)	FWHM (eV)	Peak shape	Amount (%)
Cellulose	C–O	533.0	1.55	LA*	100
100	C–O	533.0	1.73	LA	100
200	C=O	532.1	1.34	LA	48
	O–C=O	533.5	1.43	LA	52
300	C=O	532.2	1.47	LA	53
	O–C=O	533.6	1.36	LA	47

* LA- Lorentzian Asymmetric line shape.

The TGA experiments were conducted to evaluate the thermochemical properties of the hydrothermally treated cellulose. In the pyrolysis process (under pure N_2), the raw cellulose had one single mass-loss process, which started from 300–315 °C, with a sharp peak at 353 °C and ended at 360–380 °C (Supplementary Fig. 24). The pyrolysis of hydrothermal product at 100 °C was similar to the untreated cellulose. In contrast, the hydrothermal products at higher temperatures had more stable structures, making them difficult to be thermally decomposed, which might be related to the formation of the aromatic structures reflected in FTIR and Raman spectra. Two peaks at 344–358 °C and 433−500 °C could be detected in the DTG curves of hydrothermally treated cellulose from 150 °C, 200 °C, and 250 °C. However, the first peak at ca. 350 °C disappeared in the DTG

curve of the hydrochar from 300 °C, indicating the complete decomposition of hydroxy groups and six-member pyran rings in cellulose. Similar to pyrolysis, the DTG curves of cellulose combustion had only one peak, and that of hydrothermal products had two or three peaks, suggesting the transformation from the original cellulose structure to aromatic structures and fixed carbon during the hydrothermal carbonization.

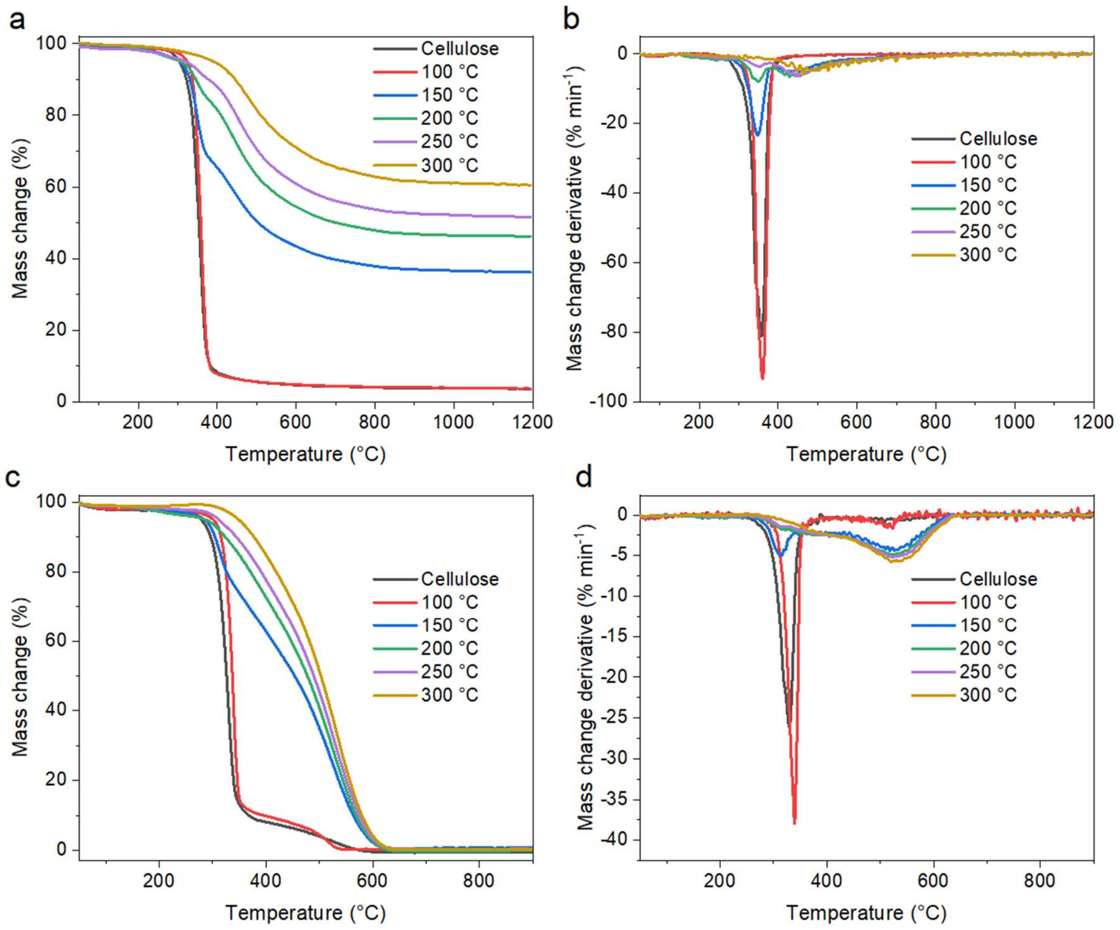

Supplementary Figure 24 | The thermogravimetric study of hydrothermally treated cellulose from different stages of the hydrothermal treatment of cellulose under 20 MPa. (a) TG curves under N_2; (b) DTG curves under N_2; (c) TG curves under air; (d) DTG curves under air.

The pyrolysis products of the hydrothermally treated cellulose were investigated online by FTIR to understand the reactions in TGA (Supplementary Fig. 25). The main products from cellulose pyrolysis were CO_2 (2360 cm^{-1}), CO (2173 cm^{-1}), H_2O (1330–1800 and 3500–4000 cm^{-1}), alkyls (2821 cm^{-1}), carboxyls (1051 cm^{-1}), and carbonyls (1745 cm^{-1}), which were directly related to a large number of hydroxyl and ether bonds in cellulose[8]. In contrast, no carboxyls or carbonyls were observed from the pyrolysis of hydrochar from 300 °C, indicating the destruction of the inherent structure (the cleavage of hydroxyl and ether bonds)[8, 9]. Interestingly, alkenyls could be detected in the FTIR, suggesting the double bonds in the hydrochar.

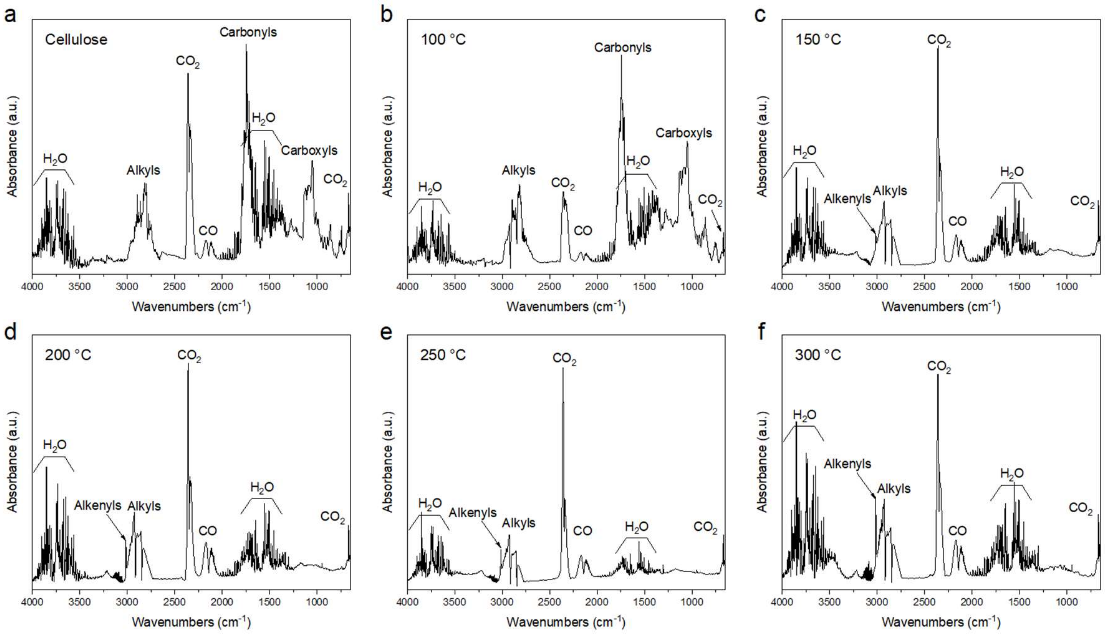

Supplementary Figure 25 | FTIR spectra of the pyrolysis products of hydrothermally treated cellulose from different stages (20 MPa) at the maximum mass loss temperature. (a) Original cellulose (353 °C); (b) hydrothermally treated cellulose from 100 °C (358 °C); (c) hydrothermally treated cellulose from 150 °C (433 °C); (d) hydrothermally treated cellulose from 200 °C (444 °C); (e) hydrothermally treated

cellulose from 250 °C (465 °C); (f) hydrothermally treated cellulose from 300 °C (500 °C). The maximum mass loss temperatures are given in parentheses.

Supplementary Note 6. Kinetics in TGA.

For pyrolysis and combustion experiments in TGA, the kinetics were calculated using the peak analysis-least square method (PA-LSM)[10]. In the parallel reaction kinetic model, the reaction was regarded as the linear combination of a series of independent reactions[11]. With each peak in the DTG curve representing an independent reaction, the whole reaction was divided into several reactions by peak analysis (PA). The kinetics of each reaction are expressed as[6]:

$$\frac{d\alpha_i}{d\tau} = k_i(1-\alpha_i)^{n_i}$$

$$k_i = A_i \exp(-\frac{E_i}{RT})$$

In TGA experiments, the heating rate β was constant, rearranging equations:

$$\frac{d\alpha_i}{dT} = \frac{A_i}{\beta}\exp(-\frac{E_i}{RT})(1-\alpha_i)^{n_i}$$

The least-square method (LSM) was used to obtain the E_i, A_i, and n_i:

$$S = \sum_{j=1}^{N}[(d\alpha/dT)_j^{\exp} - (d\alpha/dT)_j^{cal}]^2$$

where N is the number of data; $(\mathrm{d}\alpha/\mathrm{d}T)_{\exp}$ is the experimental result; $(\mathrm{d}\alpha/\mathrm{d}T)_{\mathrm{cal}}$ is the calculation result. Average deviation index (ADI) was used to evaluate the discrepancy between the experimental and calculation results:

$$\mathrm{ADI} = \frac{\sqrt{S/N}}{(\mathrm{d}\alpha_i/\mathrm{d}T)_{\max}^{\exp}} \times 100\%$$

where $(\mathrm{d}\alpha_i/\mathrm{d}T)_{\max}^{\exp}$ is the maximum among the experimental data.

The kinetics of pyrolysis and combustion of the hydrothermally treated cellulose

(Supplementary Tables 6 and 7) were calculated using the peak analysis-least square method (PA-LSM). The pyrolysis can be divided into two reactions, corresponding to the two peaks in DTG curves. The first reaction, related to the volatile formation, had the peak temperatures of 340–357 °C, activation energies of 288–417 kJ mol^{-1}, and reaction orders of 1.54–1.57. The second reaction had peak temperatures of 433–500 °C, activation energies of 40–57 kJ mol^{-1}, and reaction orders of 1.17–1.26. The combustion of hydrothermally treated cellulose could also be divided into two paralleled reactions. The first reaction at 329–339 °C, corresponded to the combustion of the volatiles in original cellulose, were only occurred in cellulose, solid product from 100 °C, and solid product from 150 °C. The reaction at 392–423 °C, found in solid products from 200 °C, 250 °C, and 300 °C, was likely due to the combustion of aromatic structure in the hydrochars. The last reaction at 513–537 °C was due to the combustion of the fixed carbon in the hydrochars.

Supplementary Table 6 | Kinetic parameters of pyrolysis of hydrothermally treated cellulose in TGA.

Reaction condition	Paralleled reaction	Percentage (%)	Peak temperature (°C)	E (kJ mol^{-1})	A (min^{-1})	n	ADI
Cellulose	1	100.0	353	288	2.53×10^{24}	1.54	1.91
100 °C, 20 MPa	1	100.0	358	347	1.53×10^{29}	1.56	1.92
150 °C, 20 MPa	1	35.8	345	288	5.29×10^{24}	1.57	1.80

	2	64.2	433	40	2.36×10^{2}	1.17	0.70
200 °C, 20 MPa	1	8.3	346	347	5.22×10^{29}	1.55	2.00
	2	91.7	444	47	7.92×10^{2}	1.21	0.83
250 °C, 20 MPa	1	2.0	344	417	7.12×10^{35}	1.57	1.94
	2	98.0	465	54	2.31×10^{3}	1.24	0.96
300 °C, 20 MPa	1	100.0	500	57	2.42×10^{3}	1.26	0.90
200 °C, 2 MPa	1	100.0	360	349	1.72×10^{29}	1.56	1.93
200 °C, 8 MPa	1	1.7	356	464	1.58×10^{39}	1.62	2.00
	2	98.3	453	51	1.66×10^{3}	1.22	0.92
200 °C, 14 MPa	1	4.7	343	408	1.33×10^{35}	1.58	1.87
	2	95.3	446	50	1.47×10^{3}	1.27	0.91

Supplementary Table 7 | Kinetic parameters of combustion of hydrothermally treated cellulose in TGA.

Reaction condition	Paralleled reaction	Percentage (%)	Peak temperature (°C)	E (kJ mol^{-1})	A (min^{-1})	n	ADI

Cellulose	1	100.0	329	304	2.67×10^{26}	1.52	2.13
100 °C, 20 MPa	1	93.6	339	452	6.96×10^{38}	1.56	2.09
	2	6.4	513	329	6.24×10^{21}	1.48	2.30
150 °C, 20 MPa	1	27.8	312	119	1.23×10^{10}	1.38	2.18
	2	72.2	528	80	3.80×10^{4}	1.52	1.32
200 °C, 20 MPa	1	45.7	392	56	3.73×10^{3}	1.32	0.96
	2	54.3	529	136	1.69×10^{8}	1.45	1.45
250 °C, 20 MPa	1	46.1	407	142	3.90×10^{8}	1.45	1.40
	2	53.9	534	61	6.77×10^{3}	1.49	1.72
300 °C, 20 MPa	1	40.3	423	138	1.91×10^{8}	1.43	1.51
	2	59.7	537	81	2.98×10^{5}	1.55	1.82
200 °C, 2 MPa	1	91.0	327	486	2.78×10^{42}	1.57	2.08
	2	9.0	480	381	1.94×10^{26}	1.50	2.30
200 °C, 8 MPa	1	39.4	342	68	1.29×10^{5}	1.27	1.96
	2	60.6	484	138	9.23×10^{8}	1.40	1.88
200 °C, 14 MPa	1	45.3	341	61	3.24×10^{4}	1.28	1.56
	2	54.7	480	162	5.09×10^{10}	1.45	1.75

Supplementary Note 7. ^{1}H NMR for hydrochar in H_2O and D_2O.

Supplementary Table 8 | ^{1}H NMR fitting parameters for hydrochars at 200 °C and 20 MPa in H_2O and D_2O.

Solvent	Peak	Chemical shift (ppm)	Assignment	Amount (%)	Area ratio to Peak 4	Percentage of decrease (%)
H_2O	1	6.12	H in the aromatic structure	73.7	3.008	-
	2	3.63	H in the oxygenated functional groups	1.3	0.053	-
	3	0.57	H in the aliphatic structure	0.5	0.020	-
	4	0.57	H in the aliphatic structure	24.5	1.000	-
D_2O	1	6.07	H in the aromatic structure	59.9	1.528	49
	2	3.60	H in the oxygenated functional groups	0.6	0.015	72
	3	0.61	H in the aliphatic structure	0.3	0.008	60
	4	0.73	H in the aliphatic structure	39.2	1.000	-

Supplementary Note 8. Methodology of sustainability assessment.

The utilization of biomass resources has a great potential in reducing global net carbon emissions when it is used as solid fuel replacing fossil energy or for soil amendment purposes with carbon sequestration benefits. To quantify the sustainability of the DTPH carbonization conceptual biorefinery designs, on a scale-up capacity of 60,000 tonnes per year, a prospective LCA based on process simulation using Aspen Plus®v11 was applied. This approach has been widely used to quantify the environmental impacts of emerging technology innovations[12-14]. Two types of waste biomass, wastepaper sludge (WPS) rich in cellulose and agricultural residue rice straw (RS), were selected as feedstocks in the prospective scenarios. The "cradle-to-grave" system boundary of LCA includes the transportation of WPS or the collection of RS, their DTPH treatment, biogas production in AD and its usage, transportation of products, and their applications in fossil fuel substitution or soil amendment.

Process simulation. The entire DTPH carbonization and subsequent wastewater treatment processes are shown in Supplementary Fig. 22. Generally, the whole process includes DTPH carbonization (A100), anaerobic digestion (AD) and aerobic digestion (AE) (A200), biogas combustion (A300), and steam generation (A400).

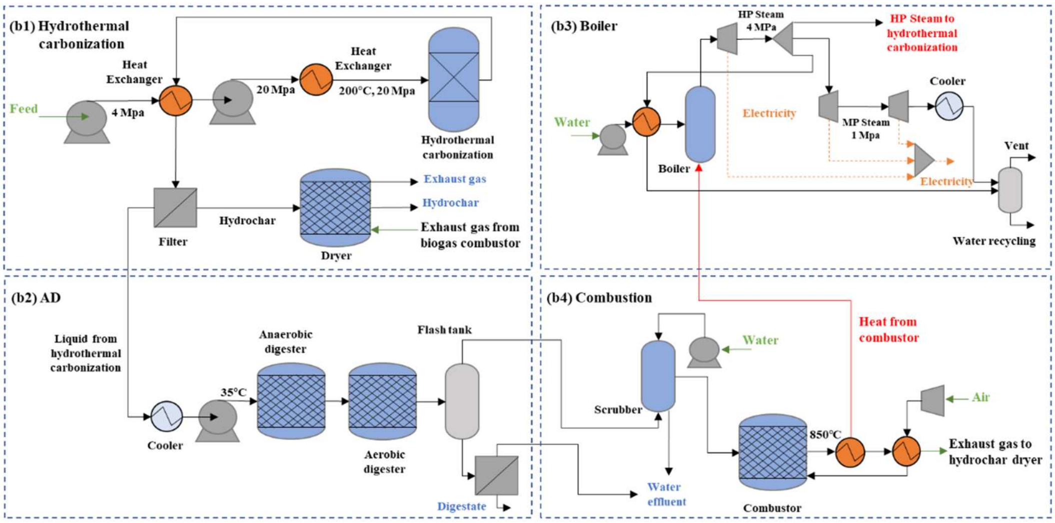

Supplementary Figure 26 | Scheme of process designs for WPS and RS DTPH carbonization biorefineries.

(1) Area 100 (A100): DTPH carbonization. Once received at the plant, the biomass feedstock is firstly treated for dedusting and size reduction prior to DTPH carbonization. Energy consumption is estimated to be 5% of the whole process[15]. Then the biomass feedstock with the reduced size is fed into the reactor, which is filled with water at 20 MPa. DTPH carbonization reactor is then heated from ambient temperature to 200 °C. Due to the complexity of reactions, a RYIELD-type reactor is chosen[16, 17]. Since the reaction pressure is over 1 MPa with hydrocarbons & solids, Peng-Robinson with Boston-Mathias (PR-BM) property method is selected for the DTPH carbonization model[17]. Experimentally obtained proximate analyses and elemental composition results of biomass feedstock (cellulose and RS) and hydrochar were used to create "nonconventional solid" components for corresponding flows in the model. According to the experiment, there is a negligible amount of gas produced. After filter separating hydrochar from the liquid, the obtained

solid is dried by the combustion outlet flue gas from the boiler to yield hydrochar with moisture content 13.6% (cellulose-derived) and 5.7% (RS-derived). No extra heat is required to keep the DTPH carbonization reaction as the reaction is exothermal[18].

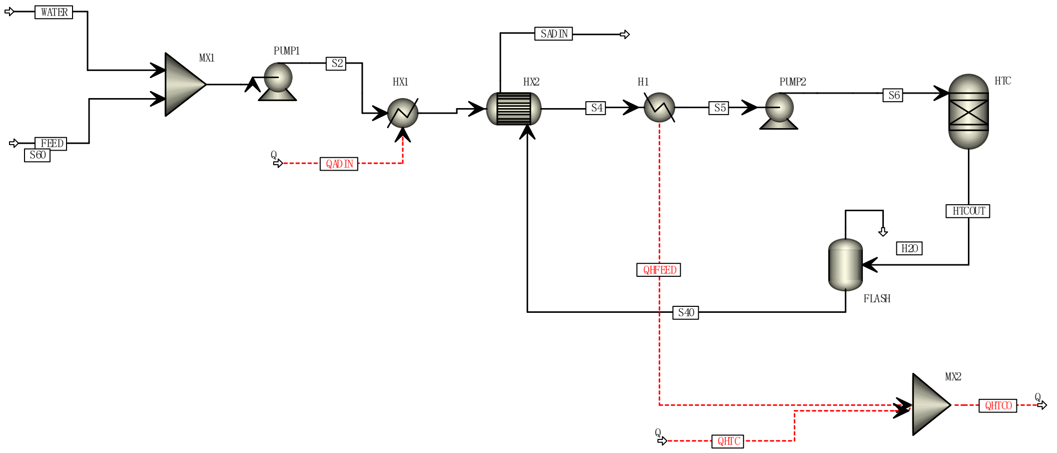

Supplementary Figure 27 | Aspen Plus simulation flowchart for DTPH carbonization (A100) section.

(2) Area 200 (A200): Anaerobic digestion (AD) and aerobic digestion (AE). Process water from DTPH carbonization is treated by AD and AE before sent to a centralized wastewater treatment (WWT) system. It is suggested that COD removal is expected to be higher than other high solid contenting wastewater stream[16]. In AD, 86% is converted to biogas (methane and carbon dioxide), and 5% is converted to cell mass. Cell mass is produced at a yield of 45 g per kg COD digested[19]. Conversion reaction equations for furfural, HMF, and other polysaccharides degradation products in DTPH carbonization were adopted from NREL process[20], so as other input materials, such as urea and other additives. Fugitive emissions from the AD were assumed to be 3.00% of the biogas produced[21], which is then sent to a scrubber for biogas cleaning. The liquid from the

anaerobic digester is pumped to aerobic activated-sludge lagoons with floating aerators. Then, 96% of the remaining soluble organic matter is removed, with 74% producing water and carbon dioxide and 22% forming cell mass. The overall COD removal achieves 99.6% after AD and AE. The mass and composition of digestate, as well as electricity consumption of dewatering, were estimated based on NREL processes[19]. The obtained digestate was assumed to be landfilled.

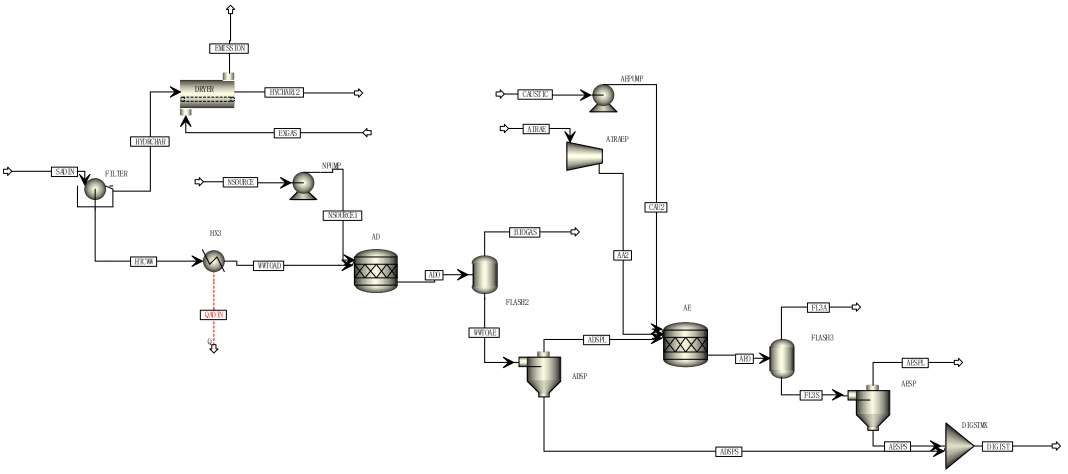

Supplementary Figure 28 | Aspen Plus simulation flowchart for AD and AE (A200) section.

(3) Area 300 (A300): Biogas combustion. The obtained biogas was first cleaned to remove NH_3 and H_2S via a scrubber which is simulated by means of RADFRAC column model without condenser or reboiler. After cleaning, biogas is burnt in a gas turbine to generate electricity. In the combustion, 10% stoichiometric excess air inlet flow is set by means of a calculator block. The boiler efficiency is assumed to be 80% based on NREL design[19]. To estimate the further reduction potential in GHG emissions via capturing and

storage of CO_2 from biogas combustion, –0.95 kg CO_2e/kg CO_2 captured was adopted considering energy used during the capture[22].

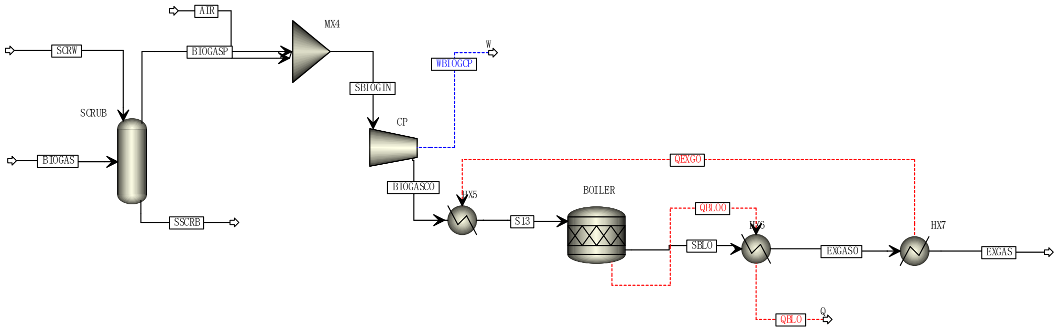

Supplementary Figure 29 | Aspen Plus simulation flowchart for biogas combustion (A300) section.

(4) Area 400 (A400): Utility. The hot exhaust gas from A300 is sent to the boiler to generate high-pressure steam which is used to heat feedstock and water before flowing to DTPH carbonization and generate electricity for pumps. The steam generated preferentially provides energy to preheat feedstocks and the remaining steam flows to turbine for generating electricity. The exhaust gas is used to dry hydrochar obtained from the press filter before discharging. Cooling water is used to take away the heat generated in reactors.

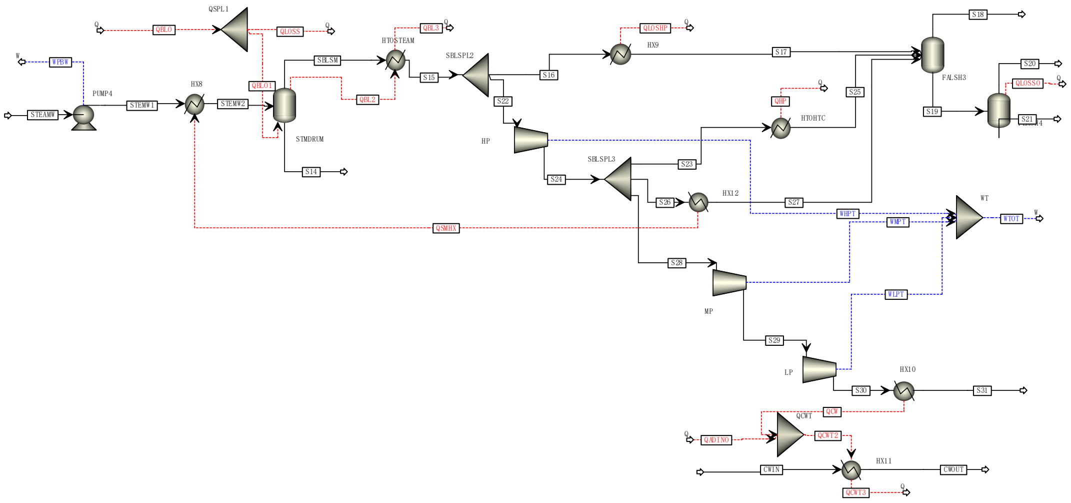

Supplementary Figure 30 | Aspen Plus simulation flowchart for boiler and utilities (A400) section.

The described processes were simulated in Aspen Plus ®V11 to generate information for the life cycle inventory. The capacity of simulation is set as 3000 L h^{-1}, corresponding to a reasonable size of DTPH carbonization reactor operated under high pressure. Process simulation specifications are listed in Supplementary Table 8.

Supplementary Table 9 | Process specifications.

	Feature	Specifications
DTPH carbonization reactor	T (°C)	200
	P (MPa)	20
Solid separator	Efficiency (%)	100
	Power (kW) [a]	0.175/kg

AD reactor [b]	T (°C)	35
	P (MPa)	0.1
	COD removal efficiency (%)	64
Biogas combustor	T (°C)	870

[a] Adopted from Aragon-Briceno et al. (2020)[16]; [b] adopted from Humbird et al. (2011)[19].

Mass and energy balances of DTPH carbonization. Supplementary Table 10 lists the proximate analyses and elemental composition results of biomass feedstock and the solid fraction of DTPH carbonization outputs. No gaseous products were identified from the experiment, and therefore, the dissolved organic carbon in liquid was estimated as TOC to close the carbon balance. Higher heating values (HHV) of feedstocks and hydrochars were calculated based on methods reported by Channiwala and Parikh[23] (Supplementary Table 6). Degradation products from polysaccharides during DTPH carbonization were estimated based on GC-MS results and kinetic models from dos Santos Rocha et al.[24] (Supplementary Data 1).

Supplementary Table 10 | Proximate analyses and elemental composition of biomass feedstocks and solid fractions of DTPH carbonization outputs.

	Proximate analysis (dry basis, wt%)			Elemental composition (dry ash-free basis, wt%)			Solid yield (%)	Carbon amount (wt%)	HHV[c] (MJ kg^{-1})
	A	V	FC	C	H	O			
Cellulose									
Feedstock	0.0	96.3	3.7	42.5	5.6	51.9			16.2
Hydrochar	0.0	53.8	46.2	70.7	4.5	24.8	43.5	72.4	27.7
Process water	0	-	-	4.70 g/L [b]	-	-			

Rice straw									
Feedstock	19.6	63.6	16.8	35.4	5	40			14.4
Hydrochar	36.2	38	25.8	41.9	3.8	18.1	50.2	59.4	17.7
Process water	0.57 g/L[a]	-	-	5.74 g/L[b]	-	-			

Note: [a] Dissolved ash in process water was measured; [b] Total organic carbon of process water was estimated. [c] HHV was calculated based on the method in Channiwala and Parikh (2002)[23]. A: ash. V: volatile matter. FC: fixed carbon.

Goal and scope of life cycle assessment (LCA). The goal of the LCA is to assess the environmental impacts of the DTPH carbonization and AD integrated technology systems using wastepaper sludge (WPS) or rice straw (RS) as feedstocks. The functional unit is the treatment of 1 tonne WPS or RS based on the composition shown in Supplementary Table 9. The WPS sludge received at the plant is assumed to contain 50 wt% water, 25 wt% cellulose, and 25 wt% ash[25]. The process simulations were performed based on a 3000 L h^{-1} reactor, and the designed plant capacity is assumed to be 60,000 tonnes per year, reflecting a biorefinery with parallel production lines. The system boundary starts with the transportation of WPS or the collection of RS, their DTPH carbonization treatment, biogas production and usage, transportation of products, and their applications. Since WPS sludge and RS are treated as waste, the environmental burdens associated with their upstream production are not included in the system boundary. For each feedstock, scenarios are set up to reflect the various utilization approaches with biomass to water ratio of feed indicated. For example, RS-SF represents the DTPH carbonization technology process of RS at various B/W ratios with hydrochar used as solid fuel. Since the fossil-based products were substituted, the system expansion allocation method was applied to avoid environmental burdens associated with the conventional products. The 2% cut-off rule was applied, and

therefore only major inputs above this threshold are included. Land-use change and infrastructure are excluded from the system.

Life cycle inventory (LCI). Mass and energy flows for cellulose are derived from our inhouse process simulation and corrected with cellulose content for WPS scenarios (Supplementary Data 1). LCA was conducted in Simapro ™ (V 9.2). For background data, the intensity of the electricity grid in Hunan Province was used in the calculation whilst relevant chemicals produced in Europe were selected from the Ecoinvent database (v 3.0) as substitutions due to the lack of information in China. GHG emissions from WPS and RS pathways were quantified via a carbon flow analysis.

In general, the transportation distance for WPS and chemicals are assumed as 100 km and 50 km via trucks, respectively. The collection for RS was calculated based on its yield. For example, Hunan as a major RS producer was taken as a place to model the conceptual plant. The amount of RS available to use in Hunan was derived from Kang et al. (2020) as 1.49 million tonnes per year[26]. The radius for collection port and transport distance was calculated and shown in Supplementary Data 1[27]. GHG reduction potential results in China were further evaluated based on RS yields in producing provinces and visualized by Geographical Information System software (ArcGIS) [26].

Life cycle impact assessment (LCIA). ReCiPe2016 (H) was applied in LCIA where six environmental impact categories were assessed, including global warming potential (GWP), stratospheric ozone depletion (ODP), terrestrial ecotoxicity (TETP), freshwater ecotoxicity (FETP), marine ecotoxicity (METP), and fossil resource scarcity (FFP).

Supplementary Note 9. Results of sustainability assessment.

The energy efficiencies for WPS sludge and RS DTPH carbonization systems at different B/W ratios are shown in Supplementary Fig. 31. The carbonization outcomes of WPS were estimated based on experimental results of cellulose DTPH carbonization. The plant *EE* on HHV basis, defined as the ratio of output energy in hydrochar and surplus electricity (if any) over input feedstock plus heat and electricity required, increase along with the enlarging B/W ratios (from 43% to 64% for WPS system and 52% to 64% for RS system).

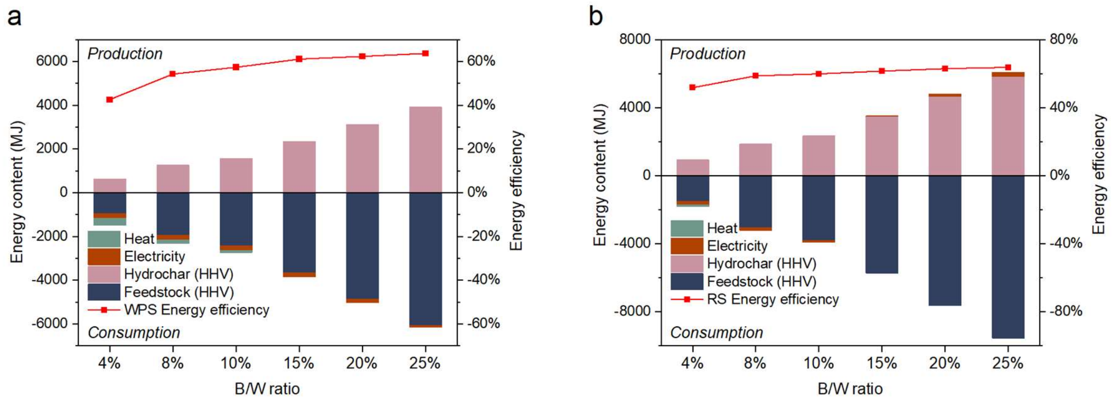

Supplementary Figure 31 | Energy efficiencies for WPS (a) and RS (b) DTPH carbonization technologies at various B/W ratios (200 °C, 20 MPa).

The mass balances of DTPH system of WPS and RS with 20 wt% B/W ratio is shown in Supplementary Table 11. These results were used in LCA analysis.

Supplementary Table 11 | Mass balances of DTPH system with 20 wt% B/W ratio for WPS and RS (200 °C, 20 MPa).

	WPS	RS
Raw material (kg h^{-1})		
Feedstock	600	600
$(NH_4)_2SO_4$	4	13
NaOH (50 wt%)	2	5
Water	3211	3425
Energy consumption (kWh)		
Electricity	37	0
Utility		
Cooling water (kg h^{-1})	707	586
Waste (kg h^{-1})		
Wastewater	3251	3363
Digestate	26	55
Process emission		
Exhaust gas (kg h^{-1})	625	1230
Product		
Hydrochar (dry, kg h^{-1})	113	284

Supplementary Note 10. Comparison between DTPH carbonization and other technologies.

To describe the carbon positive/negative potential of a technology, carbon positive/negative efficiency is proposed herein, which is defined as the total carbon in the feedstock divided by the carbon that is released or stored. The carbon positive or negative efficiency of different energy conversion technologies was then compared systemically (Supplementary Fig. 32). While biomass combustion or gasification without CCS are carbon neutral, the negative carbon efficiency of DTPH carbonization technology in this study is higher than that of biomass fermentation, comparable with biomass gasification with CCS, but lower than combustion with CCS. However, the introduction of CCS to biomass gasification or combustion will increase the capital cost and operational cost of the plant significantly, and thus these technologies are not industrially applied currently. Furthermore, the reaction temperature of DTPH carbonization (~200 °C) is lower than that of combustion (750−900 °C) or gasification (750−1150 °C).

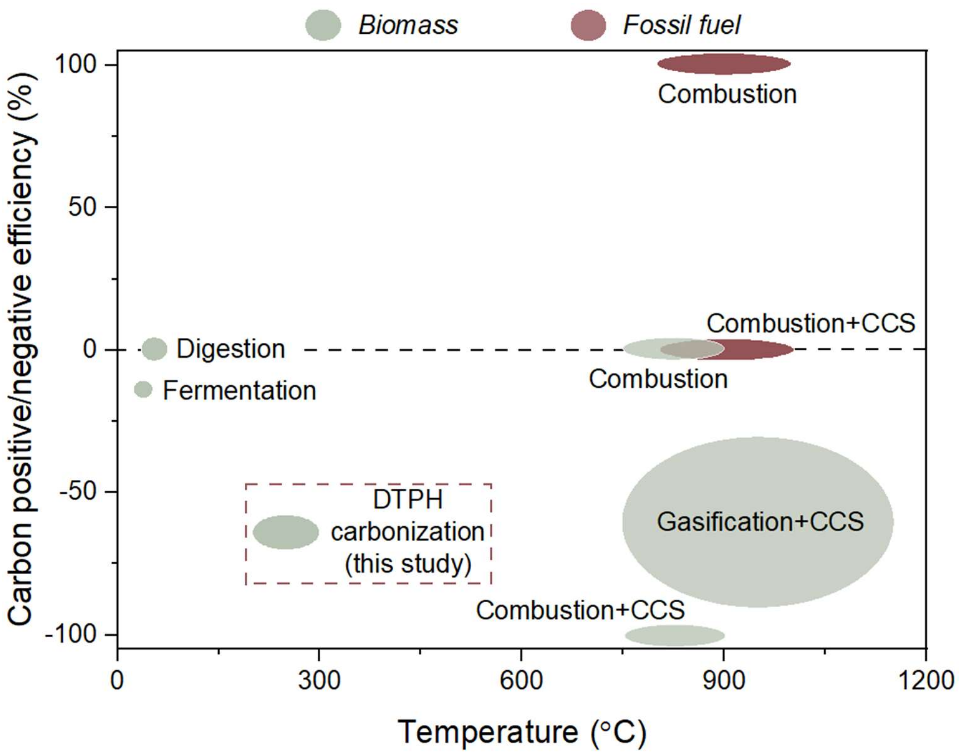

Supplementary Figure 32 | Carbon positive/negative efficiency of different energy conversion technologies with/without carbon capture and storage (CCS). The data for other technologies besides DTPH carbonization are adopted from the references[28-35].

Different NETs have different biophysical and economic limits for the target of carbon negativity. We compared the DTPH carbonization technology in this study with other NETs, including bioenergy with carbon capture and storage (BECCS), direct air capture (DAC), enhanced weathering of minerals (EW), and afforestation/reforestation (AR) (Supplementary Fig. 33). DAC, EW, and AR require less land and water; however, EW and AR are limited by the carbon-negative potential, i.e., they cannot meet the 2 °C target with the single system. DAC needs a high energy input (156 EJ yr^{-1}), which is 29% of the global energy demand[33], limiting its investment and development. The DTPH carbonization in

this study, together with BECCS, maybe one of the most potential NETs for the 2 °C target, though a significant amount of land and water are required. Therefore, it will be significant to use biomass waste, such as wastepaper sludge, agricultural waste, and forest waste, to save land and water utilization.

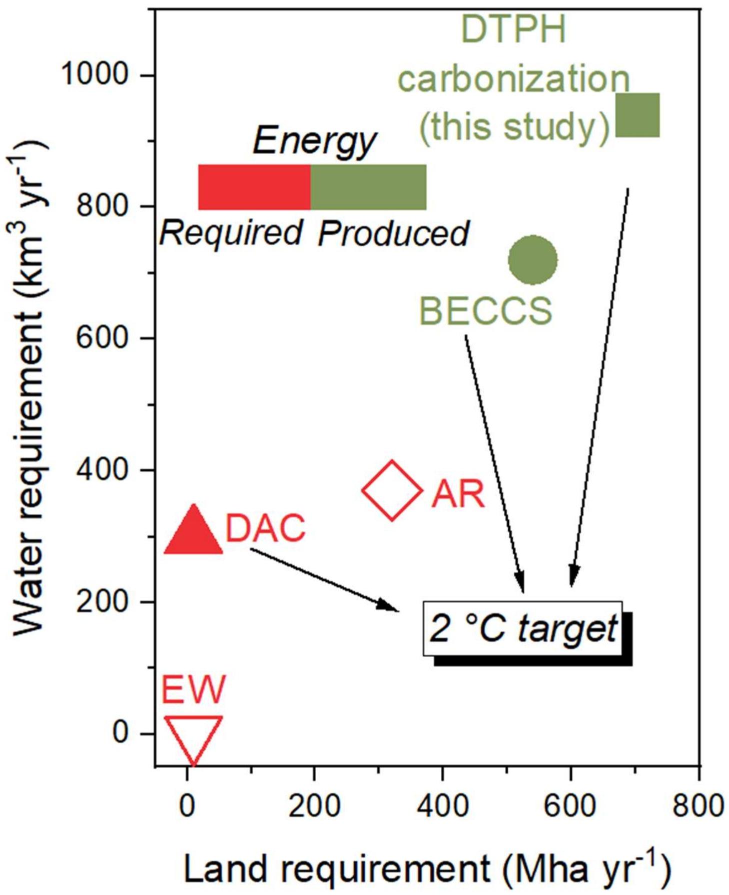

Supplementary Figure 33 | Land requirement, water requirement, energy, and carbon-negative potential of different NETs. The data for BECCS, DAC, EW, and AR are adopted from the references[28-35]. Solid shape: the method can meet the 2 °C target; hollow shape: the method cannot meet the 2 °C target.

Supplementary Note 11. Effects of other parameters on the DTPH reaction.

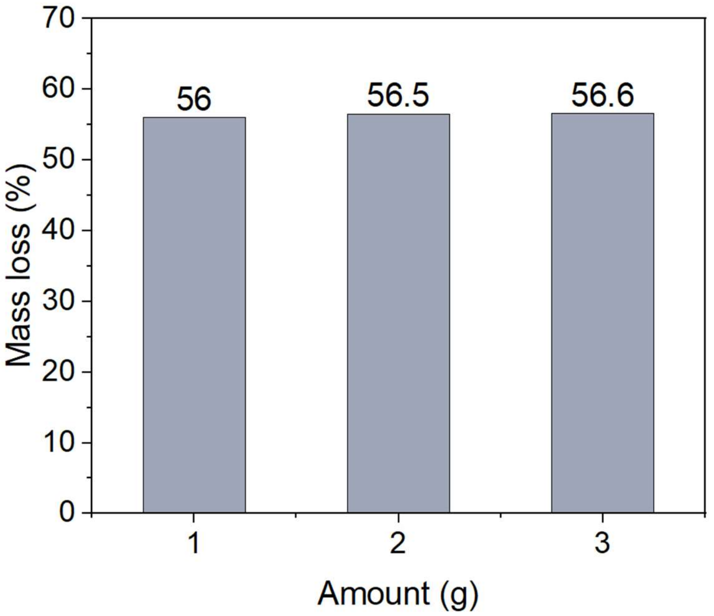

Supplementary Figure 34 | Mass loss of cellulose from different amount of starting material.

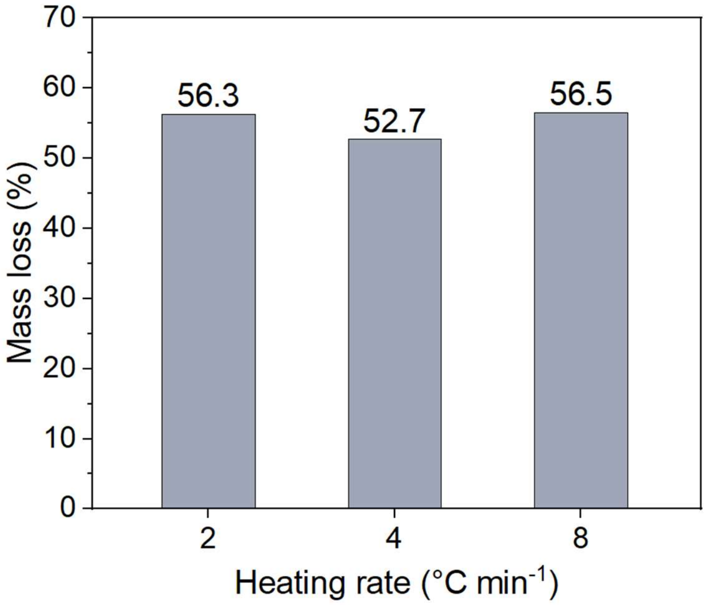

Supplementary Figure 35 | Mass loss of cellulose under different heating rates.

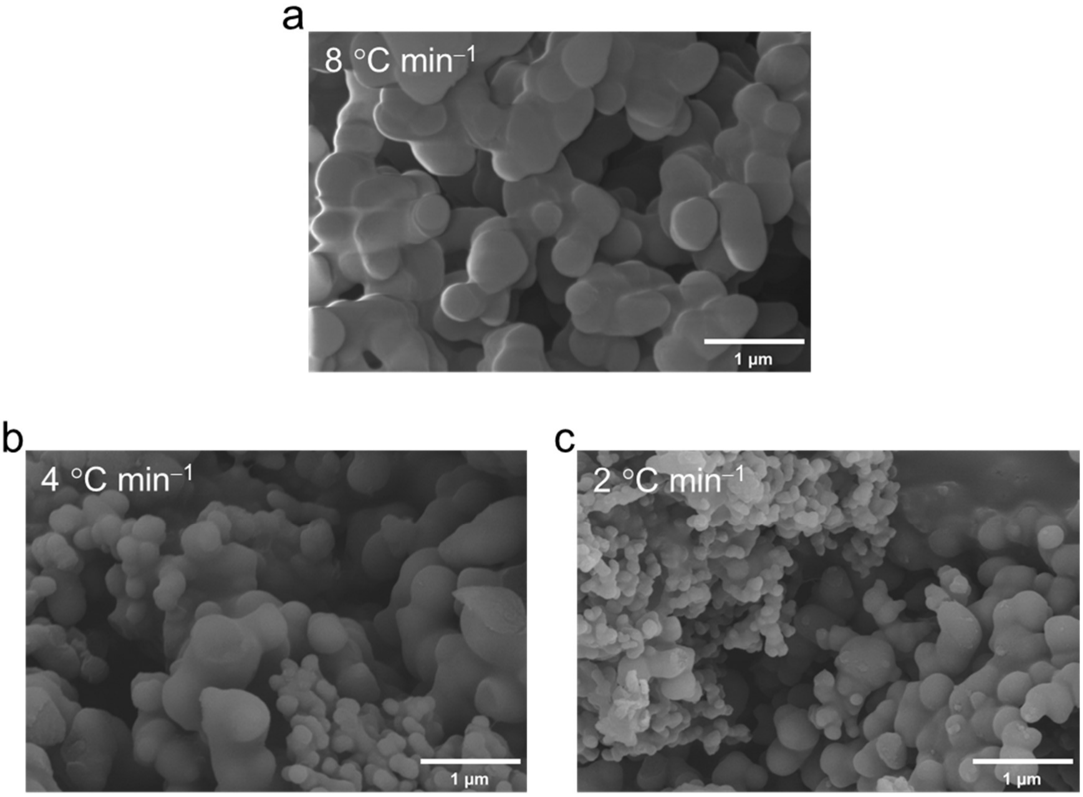

Supplementary Figure 36 | SEM of hydrochar under different heating rates.

Supplementary References

1. Sevilla M, Fuertes AB. The production of carbon materials by hydrothermal carbonization of cellulose. *Carbon* **47**, 2281-2289 (2009).

2. Falco C, Baccile N, Titirici MM. Morphological and structural differences between glucose, cellulose and lignocellulosic biomass derived hydrothermal carbons. *Green Chem.* **13**, 3273-3281 (2011).

3. Gao Y, Wang XH, Yang HP, Chen HP. Characterization of products from hydrothermal treatments of cellulose. *Energy* **42**, 457-465 (2012).

4. Kim D, Lee K, Park KY. Upgrading the characteristics of biochar from cellulose, lignin, and xylan for solid biofuel production from biomass by hydrothermal carbonization. *J. Ind. Eng. Chem.* **42**, 95-100 (2016).

5. Sheng KC*, et al.* Hydrothermal carbonization of cellulose and xylan into hydrochars and application on glucose isomerization. *J. Cleaner Prod.* **237**, 117831 (2019).

6. Coats AW, Redfern JP. Kinetic parameters from thermogravimetric data. *Nature* **201**, 68-69 (1964).

7. Sasaki M, Adschiri T, Arai K. Kinetics of cellulose conversion at 25 MPa in sub- and Supercritical water. *AlChE J.* **50**, 192-202 (2004).

8. Hansson K-M, Samuelsson J, Tullin C, Åmand L-E. Formation of HNCO, HCN, and NH3 from the pyrolysis of bark and nitrogen-containing model compounds. *Combust. Flame* **137**, 265-277 (2004).

9. Shafizad.F, Fu YL. Pyrolysis of cellulose. *Carbohydr. Res.* **29**, 113-122 (1973).

10. Zhou H, Long YQ, Meng AH, Chen S, Li QH, Zhang YG. A novel method for kinetics analysis of pyrolysis of hemicellulose, cellulose, and lignin in TGA and macro-TGA. *RSC Adv.* **5**, 26509-26516 (2015).

11. Manyà JJ, Velo E, Puigjaner L. Kinetics of biomass pyrolysis: a reformulated three-parallel-reactions model. *Ind. Eng. Chem. Res.* **42**, 434-441 (2003).

12. Yang Q*, et al.* Prospective contributions of biomass pyrolysis to China's 2050 carbon reduction and renewable energy goals. *Nat. Commun.* **12**, 1698 (2021).

13. Tian XY, Stranks SD, You FQ. Life cycle assessment of recycling strategies for perovskite photovoltaic modules. *Nat. Sustain.* **4**, 821-829 (2021).

14. Xia QQ*, et al.* A strong, biodegradable and recyclable lignocellulosic bioplastic. *Nat. Sustain.* **4**, 627-635 (2021).

15. Zhao XB, Liu DH. Multi-products co-production improves the economic feasibility of cellulosic ethanol: A case of Formiline pretreatment-based biorefining. *Applied Energy* **250**, 229-244 (2019).

16. Aragon-Briceno CI, Grasham O, Ross AB, Dupont V, Camargo-Valero MA. Hydrothermal carbonization of sewage digestate at wastewater treatment works:

Influence of solid loading on characteristics of hydrochar, process water and plant energetics. *Renewable Energy* **157**, 959-973 (2020).

17. Medina-Martos E, Istrate IR, Villamil JA, Galvez-Martos JL, Dufour J, Mohedano AF. Techno-economic and life cycle assessment of an integrated hydrothermal carbonization system for sewage sludge. *J. Cleaner Prod.* **277**, 122930 (2020).

18. Pecchi M, Patuzzi F, Basso D, Baratieri M. Enthalpy change during hydrothermal carbonization of biomass: a critical review. *J. Therm. Anal. Calorim.* **141**, 1251-1262 (2020).

19. Humbird D*, et al.* Process design and economics for biochemical conversion of lignocellulosic biomass to ethanol: dilute-acid pretreatment and enzymatic hydrolysis of corn stover.). National Renewable Energy Lab.(NREL), Golden, CO (United States) (2011).

20. Davis R*, et al.* Process design and economics for the conversion of lignocellulosic biomass to hydrocarbons: dilute-acid and enzymatic deconstruction of biomass to sugars and biological conversion of sugars to hydrocarbons.). National Renewable Energy Lab.(NREL), Golden, CO (United States) (2013).

21. Yoshida H, Monster J, Scheutz C. Plant-integrated measurement of greenhouse gas emissions from a municipal wastewater treatment plant. *Water Res.* **61**, 108-118 (2014).

22. Muller LJ*, et al.* The carbon footprint of the carbon feedstock CO2. *Energy Environ. Sci.* **13**, 2979-2992 (2020).

23. Channiwala SA, Parikh PP. A unified correlation for estimating HHV of solid, liquid and gaseous fuels. *Fuel* **81**, 1051-1063 (2002).

24. Rocha M, Pratto B, de Sousa R, Almeida R, da Cruz AJG. A kinetic model for hydrothermal pretreatment of sugarcane straw. *Bioresour. Technol.* **228**, 176-185 (2017).

25. Frías M, Vegas I, de la Villa RV, Giménez RG. Recycling of waste paper sludge in cements: characterization and behavior of new eco-efficient matrices. In: *Integrated Waste Management-Volume II*). IntechOpen (2011).

26. Kang YT*, et al.* Bioenergy in China: Evaluation of domestic biomass resources and the associated greenhouse gas mitigation potentials. *Renew. Sustain. Energ. Rev.* **127**, 109842 (2020).

27. Liu F, Dong XY, Zhao XB, Wang L. Life cycle assessment of organosolv biorefinery designs with the complete use of biomass. *Energy Convers. Manage.* **246**, 114653 (2021).

28. Klein D*, et al.* The value of bioenergy in low stabilization scenarios: an assessment using REMIND-MAgPIE. *Clim. Change* **123**, 705-718 (2014).

29. Schuiling RD, Krijgsman P. Enhanced weathering: an effective and cheap tool to sequester co2. *Clim. Change* **74**, 349-354 (2006).

30. Smith LJ, Torn MS. Ecological limits to terrestrial biological carbon dioxide removal. *Clim. Change* **118**, 89-103 (2013).

31. Stolaroff JK, Keith DW, Lowry GV. Carbon dioxide capture from atmospheric air using sodium hydroxide spray. *Environ. Sci. Technol.* **42**, 2728-2735 (2008).

32. Bonsch M*, et al.* Trade-offs between land and water requirements for large-scale bioenergy production. *GCB Bioenergy* **8**, 11-24 (2016).

33. Smith P*, et al.* Biophysical and economic limits to negative CO2 emissions. *Nat. Clim. Change* **6**, 42-50 (2015).

34. Sanchez DL, Kammen DM. A commercialization strategy for carbon-negative energy. *Nat. Energy* **1**, 15002 (2016).

35. Haberl H*, et al.* Quantifying and mapping the human appropriation of net primary production in earth's terrestrial ecosystems. *Proc. Natl. Acad. Sci.* **104**, 12942 (2007).
