# Supplementary material for: Decoupled temperature and pressure hydrothermal synthesis of carbon sub-micron spheres from cellulose
Source: Nat Commun. 2022 Jun 24;13:3616. doi: 10.1038/s41467-022-31352-x (PMC9232491; doi:10.1038/s41467-022-31352-x)
Supplement: Supplementary file 3 — Description of Additional Supplementary Files [file 41467_2022_31352_MOESM3_ESM.pdf]

Description of Additional Supplementary Files

File Name: Supplementary Data 1
Description: **Detailed data of life cycle assessment.**
Sheet A: Liquor component
Sheet B: Energy Efficiency
Sheet C: Transport
Sheet D: LCI
Sheet E: Coal substitution LCIA
Sheet F: Soil amendment LCIA
Sheet G: RS GWP China
